# Beyond Probiotics: Postbiotics for Healthy Aquaculture

**DOI:** 10.1002/advs.77737

**Published:** 2026-09-27

**Authors:** Michael Leonidas Chikindas, Delin Xu, Igor V. Popov, Kayque Ordonho Carneiro, Nilufar Arashovna Elova, Gullola Amirovna Bekmurodova, Dildora Amindjanovna Amirsaidova, Astghik Pepoyan, Shakhlo Mirjamalovna Miralimova, Oleg V. Mitrokhin, Liubov S. Sichel, John Tagg, Svetoslav Dimitrov Todorov

**Affiliations:** ^1^ Health Promoting Naturals School of Environmental and Biological Sciences Rutgers State University New Brunswick New Jersey USA; ^2^ Department of General Hygiene Sechenov First Moscow State Medical University Moscow Russia; ^3^ Department of Ecology Institute of Hydrobiology School of Life Science and Technology at Jinan University Guangzhou China; ^4^ Institute of Life Science Don State Technical University Rostov‐on‐Don Russia; ^5^ ProBacLab Laboratório De Microbiologia de Alimentos Departamento De Alimentos e Nutrição Experimental Food Research Center Faculdade de Ciências Farmacêuticas Universidade De São Paulo São Paulo São Paulo Brazil; ^6^ Institute of Microbiology of the Academy of Sciences of the Republic of Uzbekistan Tashkent Uzbekistan; ^7^ Department of Food Safety and Biotechnology Armenian National Agrarian University Yerevan Armenia; ^8^ Stellar Biotics LLC Rockleigh New Jersey USA; ^9^ BLIS Technologies Ltd. South Dunedin Dunedin New Zealand; ^10^ CISAS‐ Center for Research and Development in Agrifood Systems and Sustainability Instituto Politécnico de Viana do Castelo Viana do Castelo Portugal

**Keywords:** antimicrobial resistance, aquaculture, sustainable aquaculture, aquafeeds, biosafety, fish health management, gut microbiota dysbiosis, probiotics, postbiotics

## Abstract

With a growing global population and strict restrictions on traditional antibiotics, sustainable fish health management is crucial for aquaculture. While live probiotics are popular alternatives, their real‐world effectiveness remains highly controversial. Variable effectiveness results from complex interactions with different fish species, differences in fish gastrointestinal anatomy, and variations in environmental parameters such as salinity, pH, and temperature. Furthermore, live probiotics pose risks of ecological disturbance and horizontal gene transfer of antimicrobial resistance. This critical review assesses these limitations and proposes postbiotics, preparations of inanimate microorganisms and their metabolites, as a more stable, controlled, and biosafe formulation. Postbiotics offer significant advantages during industrial aquafeed production and storage. However, their practical implementation is currently hindered by vague terminology and legislative gaps. To overcome these obstacles, this review introduces an innovative modular analytical model utilizing specific chemical biomarkers (such as short‐chain fatty acids, bacteriocins, and lipoteichoic acids). Implementing these matrix‐matched, multi‐module quality control panels to verify molecular safety, identity, and efficacy is critical to standardizing next‐generation “precision postbiotics” for sustainable aquaculture development.

## Graphical abstract

This critical review assesses limitations of probiotics and proposes postbiotics, preparations of inanimate microorganisms and their metabolites, as a more stable, controlled, and biosafe formulation. Postbiotics offer significant advantages during industrial aquafeed production and storage. However, their practical implementation is currently hindered by vague terminology and legislative gaps.

## 1 Introduction

One of the most important developments emerging from probiotic research is the concept of postbiotics. As a next step in the use of beneficial microbes, postbiotics involve non‐viable microbial cells and their metabolites, which can be used to support the health of farm animals, pets, and humans [1].

For centuries, aquaculture has been part of the food industry (brilliantly reviewed by Nash [2]). However, since the last century, due to population growth and limitations of land‐based farming methods, more attention has been paid to the production of proteins from aquatic organisms, taking care of sustainability and the circular economy [3]. Furthermore, the overexploitation of natural aquatic resources has been recognized as a flawed practice, and today, controlled aquaculture development is part of the UN Directive on Responsible Food Production (see the FAO Code of Conduct for Responsible Fisheries [4]), based on the philosophy of the One Health initiative [5].

The intensification of aquaculture has increased the need for effective and environmentally responsible aquatic health management strategies [6]. Thus, probiotics, defined as live microorganisms that, when administered in adequate amounts, confer a health benefit on the host [7], have become a key component in the search for sustainable alternatives in aquaculture and improving yields using existing natural resources [8, 9]. However, despite extensive research and commercial enthusiasm, the results of using probiotics in aquaculture remain controversial due to the specific nature of farming aquatic animals. Many studies report positive effects, while others show little or negative impact, including on the environment and conservation—topics that will be critically addressed in this review. Moreover, there is growing concern among aquaculture farmers about the effectiveness of probiotics in aquaculture practices. These discrepancies raise important questions about the reliability of probiotic interventions and the assumptions underlying their widespread adoption, especially when it concerns aquaculture (critically reviewed by Todorov et al. [10]).

In this review, the reader's attention is drawn to the complexity and differences in the anatomy, physiology, and habitat of different fish, which makes it impossible to use the same probiotics with the same effectiveness for different fish species from different habitats. A critical analysis of the limitations of the effective use of probiotics in aquaculture leads to the suggestion that these limitations can be overcome by introducing postbiotics that can be applied to a wider range of aquaculture species and in wider habitat conditions. This is probably realistic, since postbiotics, while capable of exerting a positive effect on a eukaryotic organism comparable to the effect of probiotics, are formulations derived from non‐purified preparations of inanimate microorganisms [11].

To search and critically assess the literature published up to and including March 2026, PubMed, Web of Science, and Scopus were used using combinations of keywords: probiotics; postbiotics; aquaculture; fish health management; gut microbiota dysbiosis; antimicrobial resistance; biosecurity; aquaculture feed; sustainable aquaculture. Unlike previous reviews that primarily catalogue applications of postbiotics in aquaculture, this review develops a conceptual framework explaining the transition from probiotics to postbiotics through the lens of environmental robustness, formulation standardization, and precision microbial interventions.

## 2 Chapter 1. Differences in Intestinal Microbiota of Various Fish Species and In Their Habitats

### 2.1 Interplay Between Host Species, Habitat, and the Fish Microbiome

Intestinal microbiota of fish have co‐evolved with their hosts and play a critical role in nutrient digestion, immune system development, and pathogen resistance [12, 13, 14]. Due to the remarkable diversity of fish in habitat, diet, anatomy, and physiology, the factors that shape their associated microbial consortia have become a central focus of study. This section summarizes current knowledge on variations in microbiota composition across different fish species and how these communities are dynamically influenced by the diverse ecological niches of their hosts, focusing primarily on studies from the last five years while also incorporating key insights from the past decade.

### 2.2 Influence of Host Species on Gut Microbiota

#### 2.2.1 Trophic Level

The gut microbiota of fish is predominantly composed of the phyla Proteobacteria, Firmicutes, Bacteroidetes, and Actinobacteria, with Proteobacteria often being the most abundant [15, 16]. At the genus level, common taxa include *Aeromonas*, *Pseudomonas*, *Clostridium*, Lactobacilli, and *Bifidobacterium* [17]. Host species and their feeding habits significantly shape the composition, diversity, and function of fish gut microbiota [18, 19]. Multiple studies consistently show that herbivorous fish exhibit higher gut microbial diversity (Shannon/Simpson indices) than carnivorous, omnivorous, or planktivorous species [20, 21, 22]. At the genus level, herbivores are enriched in polysaccharide‐degrading bacteria (e.g., *Niameyobacter*, *Alistipes*, *Akkermansia*, *Clostridium*, *Citrobacter*) and carbohydrate metabolism pathways (e.g., pyruvate, butanoate, propanoate), whereas carnivores harbor more protease‐producing bacteria (e.g., *Clostridium*, *Cetobacterium*, *Halomonas*) and enriched amino acid metabolism pathways [20, 21, 22]. The functional basis of these differences lies in the digestive requirements of each diet. Herbivorous fish lack endogenous cellulase enzymes and instead rely on gut microbes to degrade plant cell walls [19]. Such adaptive differences were also validated in three wild cold‐water fish species, which allow for efficient digestion of species‑specific diets [23]. Quantitative support comes from Liu et al. [19], who demonstrated significantly higher cellulase and amylase activities in herbivorous fish and much higher trypsin activity in carnivorous fish. This information highlights that host‑specific feeding habits drive adaptive differences in gut microbiota, enabling efficient digestion of species‑typical diets. However, the relative importance of diet versus other factors remains debated. A comprehensive study of 227 wild fish species demonstrates that host habitat (freshwater *vs*. seawater) is the primary determinant of gut microbiota composition, outweighing the influence of host taxonomy and trophic level [16]. A large‑scale study across the Yellow River (China) [24] examining 24 fish species over 1500 km found that although diet significantly influenced gut microbiota, it explained only a small fraction of variation (4.2% by Adonis; 0.8%–5% by MRM). In contrast, geographical distance (26.6%) and host evolutionary history (9.8%) were the dominant drivers, with dispersal limitation governing community assembly over large spatial scales. This discrepancy suggests that the strong diet‑associated signals observed in controlled or local‑scale studies may be overshadowed by spatial and phylogenetic factors when sampling across broad geographic ranges. Thus, the importance of diet in shaping fish gut microbiota is context‑dependent, being more pronounced at small scales or under similar environmental conditions, while large‑scale biogeographic and evolutionary processes can become primary determinants.

#### 2.2.2 Phylogeny and Host Genetics

The intestinal digesta microbiota of fish are distinct from the microbiota of the surrounding water [25]. Accumulating evidence from wild and farmed fish demonstrates that host evolutionary history and genetic background profoundly influence gut microbiota composition, yet the relative importance of phylogeny versus environmental factors varies across spatial scales and experimental contexts. Recent large‑scale studies have substantially advanced our understanding of these complex relationships.

Tang et al. [26] conducted a metagenomic analysis of 903 samples from 121 wild freshwater fish species in southwest China, constructing 705 metagenome‑assembled genomes. Host phylogeny explained 22.28% of gut α‑diversity and 48.2% of skin α‑diversity, with significant phylosymbiosis (microbiota composition correlates positively with host phylogenetic distance), particularly in species from the family Cyprinidae. In contrast, Lilli et al. [27] showed that geography overrode host phylogeny in Mediterranean scorpionfish, though a weak phylosymbiosis signal remained. Sevellec et al. [28] demonstrated that lake‑specific conditions often surpass host effects in wild whitefish, yet common‑garden experiments confirmed modest host genetic influences, with hybrid microbiota diverging from parental lines. Minich et al. [29] identified body site as the strongest driver across 101 marine fish species, with phylosymbiosis detectable in the hindgut, gill, and skin. Jin et al. [30] revealed that intrageneric hybridization in pufferfish alters gut microbiota more than cohabitation, shifting metabolic priorities toward carbohydrate utilization. Beyond genetics, Piazzon et al. [31] showed that selectively bred fast‑growing sea bream possess a more plastic microbiota and an altered intestinal transcriptome. Similarly, Naya‑Català et al. [32] found that such selected fish maintain a stable microbial community even under alternative diets, with enhanced functional plasticity linked to host lipid metabolism and epithelial regeneration. Ziab et al. [33] used hybrid Chinook salmon in a controlled common‑environment design to demonstrate that additive genetic variance within populations, particularly sire effects, contributes significantly to gut microbiota composition, revealing population‑specific heritability patterns. Anka et al. [34] leveraged inbred killifish strains to disentangle host from environmental effects, showing that host genetics (strain) and early hatching mode shape microbiota diversity, which in turn correlates with fish activity and anxiety‑like behaviors, suggesting an indirect genetic pathway via microbiota. Meanwhile, Hansen et al. [35] linked DNA methylation changes in diseased salmon to microbiota displacement. Schwob et al. [36] provided strong evidence of co‑phylogeny between Harpagifer and its core symbiont *Aliivibrio*, reinforcing that recently diverged fish models can reveal host‑microbe co‑evolution. Collectively, these studies underscore that host phylogeny, hybridization, epigenetics, and body site jointly shape fish gut microbiota, with future research needing multi‑omics and temporal sampling to disentangle causal mechanisms.

#### 2.2.3 Intestinal Anatomy

Anatomical features such as intestinal segmentation, relative length, and ontogenetic structural development create distinct physicochemical microenvironments that directly shape microbial colonization, diversity, and function [37]. Fu et al. [38] systematically compared the anterior, middle, and posterior intestines of the pufferfish (*Takifugu obscurus*) and found that mucosal fold height, muscularis thickness, and cross‑sectional area vary significantly along the gut. These morphological differences correlate with segment‑specific digestive enzyme activities and, importantly, distinct microbial communities, where the anterior and middle intestines are dominated by Epsilonbacteraeota and Proteobacteria, while the posterior segment shows enrichment of *Spirochaetes* [38]. Similarly, juvenile greater amberjack (*Seriola dumerili*) exhibits longer microvilli and higher fold heights in the anterior and posterior intestines than in the midgut, a structural heterogeneity mirrored by functional microbial zonation where *Ruminococcus* (lipid digestion) dominates the anterior gut while *Prevotella*, *Bifidobacterium*, and lactobacilli (carbohydrate metabolism and immunity) are enriched in the midgut [39]. Together, these studies demonstrate that regional differences in intestinal morphology create distinct ecological niches, leading to a predictable spatial organization of the microbiota.

Beyond regional differences, the gross anatomical feature of intestinal length exerts a strong influence on the gut microbiota. Relative intestinal length was demonstrated by Jiao et al. [40] as an important factor shaping the pattern. Among four sympatric fish species with different feeding habits (omnivorous, herbivorous, carnivorous, planktivorous), carnivorous fish had the most complex microvilli branching yet the lowest gut microbial diversity, whereas omnivorous fish showed the highest diversity [40]. In a comprehensive study on eight cyprinid species, Liu et al. [41] reported that fish with similar relative gut lengths harbor similar gut microbial communities. Species with shorter intestines are dominated by Proteobacteria with fewer Firmicutes, while longer intestines favor taxa such as *Clostridium* and *Romboutsia*. Moreover, the carbohydrate‑metabolism capacity of the microbiota correlates positively with relative gut length, whereas amino acid and lipid metabolism show negative correlations [42]. These findings indicate that intestinal length is not merely a passive trait but actively filters microbial membership and programs the metabolic potential of the resident community.

A study on juvenile *Procypris mera* shows that gut anatomical maturation, marked by intestinal gland appearance at 50 days post‑hatching, shapes microbiota assembly [43]. Microbial diversity fluctuates with Proteobacteria and Firmicutes dominant, and the progressive differentiation of the epithelium provides expanding colonization surfaces [43]. This temporal coupling suggests host developmental programs dictate microbiota assembly.

The presence of a stomach (as in many carnivores) creates an acidic foregut, which filters environmental microbes, leading to stochastic intestinal colonization. In contrast, stomachless fish allow direct microbial input from the environment into the gut, which renders their gut microbiota more susceptible to ecological selection and dispersal processes [44]. The ecological consequences are evident in microbiota composition and diversity. Studies on agastric species such as the Mexican pike silverside (*Chirostoma estor*) reveal that their intestinal microbiota is dominated by Firmicutes and Proteobacteria, but with significantly lower diversity in cultured individuals compared to wild ones, highlighting the strong influence of habitat and diet in the absence of gastric filtering [45]. Furthermore, the lack of a stomach may drive functional compensation via the gut microbiota. Research on the agastric common carp shows that individuals feeding on natural plankton exhibit higher digestive enzyme activities (e.g., protease, chitinase) compared to cultured counterparts, suggesting that the intestinal microbiota in stomachless fish may play an enhanced role in breaking down complex polysaccharides [46]. From an applied perspective, the reduced microbial barrier in agastric fish makes them more responsive to dietary interventions, such as probiotics and prebiotics, which can effectively modulate their gut microbiota and improve health outcomes, an advantage less pronounced in stomach‐bearing species with a more stable, acid‐filtered community [37]. Collectively, these findings underscore that gut anatomy is not merely a structural trait but a primary determinant of host‐microbe interactions, dictating how environmental microbes colonize the gut and how the microbiota contributes to host digestion and health.

### 2.3 Influence of Habitat Environmental Factors on Gut Microbiota

#### 2.3.1 Salinity

Salinity represents one of the most fundamental environmental gradients shaping fish gut microbiota. Diverse meta‐analyses identified salinity as a primary factor distinguishing gut bacterial communities across fish species [47]. Freshwater and marine fish harbor distinct core microbiota: freshwater communities are often enriched in *Aeromonas*, *Mycoplasma*, *Clostridium*, and *Cetobacterium*, while marine fish show higher abundances of *Vibrio*, *Pseudoalteromonas*, *Paracoccus*, and *Photobacterium* [48]. Ofek et al. [21] suggested that the genus *Cetobacterium* consistently occupies a core position in the intestines of various freshwater fish, while it is almost undetectable in marine fish.

Salinity stress typically reduces gut microbial diversity when fish are reared outside their optimal range. In Nile tilapia, freshwater‐reared fish exhibited higher microbial diversity than those in high salinity (salinity around 25 ppt) [49]. Similarly, yellowfin seabream (*Acanthopagrus latus*) in seawater (30 ppt) had significantly higher gut microbial richness (Chao1) and diversity (Shannon) compared to brackish water (10 ppt) [50]. In contrast, during progressive salinity acclimation of rainbow trout (*Oncorhynchus mykiss*), alpha diversity dropped sharply once salinity exceeded 0 ppt, then stabilized, while seawater‐adapted individuals developed a simpler but more stable co‐occurrence network [51]. These findings suggest that salinity stress filters salt‐sensitive taxa and selects for tolerant species, driving the microbiota toward a simplified but resilient state.

One of the most consistent patterns is the dominance of *Cetobacterium* (phylum Fusobacteria) in freshwater or low‐salinity environments, whereas *Vibrio* (Proteobacteria) proliferates under high salinity. In Nile tilapia, a 5% dietary NaCl supplement combined with 25 ppt water salinity significantly increased *Cetobacterium* abundance, which correlated with improved growth and anti‐inflammatory gene expression [50]. In yellowfin seabream (*A. latus*), *Cetobacterium* accounted for 72% of the gut microbiota in brackish water (10 ppt) and positively correlated with host metabolic genes [50]. When Asian sea bass (*Lates calcarifer*) were transferred from seawater to freshwater, *Cetobacterium* and *Plesiomonas* became dominant, while pathogenic genera such as *Vibrio*, *Staphylococcus*, and *Acinetobacter* declined [52]. Conversely, seawater‐enriched *Vibrio* (31.5% in yellowfin seabream) and *Candidatus arthromitus*, and these shifts were associated with upregulation of pro‐inflammatory cytokines [50]. In grass carp (*Ctenopharyngodon idella*), high salinity (6 ppt) led to replacement of *Cetobacterium* by *Vibrio*, *Aeromonas*, and *Pseudomonas*, accompanied by growth inhibition [53].

Salinity‐driven microbiota changes are tightly linked to host physiology. Brackish/freshwater‐adapted fish exhibit gut microbiota enriched in carbohydrate, vitamin, and amino acid metabolism, as well as DNA repair pathways, while seawater‐adapted fish show enhanced cell motility and immune‐related pathways [50, 54]. Notably, in rainbow trout, seawater acclimation induced DNA damage and upregulation of cancer risk‐related genes in host tissues, which correlated with low‐abundance gut bacteria such as *Psychrobacter celer* and lactobacilli, suggesting a “gut‐organ axis” involvement in salinity adaptation [51]. Furthermore, salinity alters microbial community assembly processes, with homogenizing dispersal dominating in freshwater rainbow trout, whereas stochastic drift becomes predominant in seawater [51].

#### 2.3.2 Temperature and Seasonality

Ectothermic fish are uniquely vulnerable to temperature fluctuations. Given climate change‐induced gradual warming and more frequent heatwaves, recent studies have increasingly explored how thermal and seasonal changes affect fish‐associated microbial communities. This section synthesizes the past five years' findings on temperature and seasonality effects on gut microbiota composition, diversity, stability, and function.

Across multiple fish species, water temperature profoundly alters gut microbial community structure, though responses are species‐specific and temperature‐dependent. In Nile tilapia (*Oreochromis niloticus*), +2°C warming causes transient, recoverable changes, whereas +8°C leads to persistent dysbiosis [55]. In rainbow trout (*O. mykiss*), heat stress (24°C) alters Firmicutes, Gemmatimonadetes, and Bacteroidetes; acute heat reduces diversity, promotes *Mycoplasma* and Firmicutes, and suppresses beneficial microorganisms like lactobacilli [55, 56], while 18°C yields higher diversity than 14°C or 20°C, enhancing propanoate metabolism [57]. In European seabass (*Dicentrarchus labrax*), temperature stress shifts *Faecalibacterium*, *Filifactor*, *Butyricicoccus*, and Erysipelotrichaceae UCG‐006, alongside changes in amino acid and lipid metabolites [41, 42]. In golden pompano (*Trachinotus blochii*), low temperature (18°C) elevates serum AST, ALT, and triglycerides, and enriches *Mycoplasma*, *Vibrio*, and *Spirochaeta* [58]. In largemouth bass (*Micropterus salmoides*), acute temperature fluctuations decrease Firmicutes and Verrucomicrobiota, but increase Fusobacteria, Actinobacteria, and Proteobacteria [59]. Recovery capacity following thermal disturbance is species‑specific. Nile tilapia (*O. niloticus*) exhibit strong resilience to +2°C warming, with microbial communities returning to baseline, but fail to recover from +8°C, where long‑term warming disrupts network stability [55]. Temperature also influences the ecological processes governing microbial assembly. In chum salmon (*Oncorhynchus keta*), deterministic (environmental selection) dominates gut community assembly, whereas stochastic processes shape skin microbiota [60]. Temperature can drive distinct compositional replacements with functional implications. In Chinook salmon (*Oncorhynchus tshawytscha*), warming replaces *Photobacterium* (Vibrionaceae) with *Cetobacterium* (Fusobacteriaceae) and *Brevinema* [61]. Notably, *Cetobacterium* correlates positively with elevated temperature and may contribute to vitamin B12 production and immune modulation [62].

The magnitude and duration of temperature change critically determine outcomes. In kaluga sturgeon (*Huso dauricus*), Wang et al. [63] observed that short‐term high‐temperature stress initially increased microbial richness, followed by gradual stabilization. Notably, heat‐resistant specimens exhibited a higher abundance of beneficial *Cetobacterium*, while heat‐stressed fish harbored significantly more *Plesiomonas*, a known opportunistic pathogen. In hybrid sturgeon, Yang et al. [64] reported that extreme heat (28°C) caused an explosive increase in thermophilic pathogens, including *Plesiomonas*, *Cetobacterium*, and *Aeromonas*, accompanied by severe enteritis and suppression of digestive enzyme activities.

Different fish species exhibit markedly different thermal sensitivities. In Atlantic salmon (*Salmo salar*) farmed in warm Tasmanian waters, Reid et al. [65] showed that *Aliivibrio*, *Vibrio*, and *Photobacterium* dominate the gut mucosal layer, with peak abundance occurring 7–8 months after seawater transfer during late summer. These Vibrioaceae species possess diverse colonization‐associated genes, including those for biofilm formation, mucin degradation, and, in some strains, cytolethal distending toxin, suggesting that temperature‐driven shifts may have virulence implications [65].

Tissue compartment also matters. In rainbow trout (*O. mykiss*), Zhou and Ding [56] found that heat stress exerted the most pronounced effects on gill and skin mucus microbiota, followed by gastrointestinal digesta, while gastrointestinal mucosa showed relatively minor changes. In sturgeon (family *Acipenseridae*), Yang et al. [63] demonstrated that heat stress caused severe damage to the valve intestine specifically, with mitochondrial enlargement, endoplasmic reticulum dilation, and inflammatory cell infiltration, correlating with overgrowth of pathogenic bacteria in the intestinal microbiota.

Field studies tracking fish across seasons provide critical real‐world validation. In red‐spotted grouper (*Epinephelus akaara*), Sun et al. [66] observed that both captive and wild populations exhibit the highest gut microbial diversity in winter, contrasting with patterns seen in temperate species where summer peaks are typical. Season explained over 19% of bacterial community variation in pangasius ponds and 16% in tilapia ponds in Bangladesh [66], with temperature and pH emerging as significant environmental correlates. Core taxa, including *Pseudomonas* (phylum Pseudomonadota) as well as members of the phyla Planctomycetota and Actinomycetota, persist across seasons, but their relative abundances shift dramatically, with *Cyanobacteria* blooming in warmer months.

Temperature stress‐induced gut dysbiosis compromises fish health. In Tsinling lenok trout (*Brachymystax lenok*), high temperature (24°C) reduced intestinal villus height, epithelial cell thickness, and antioxidant enzyme activities (SOD, GSH‐Px), while increasing MDA content; metabolomics revealed decreases in glutathione, adenosine, and other metabolites, along with increased D‐glucose‐6‐phosphate, indicating oxidative stress and impaired DNA/RNA synthesis [67]. In largemouth bass, acute temperature fluctuations activated antioxidant systems, but cold and heat stresses induced distinct metabolic responses. Cold stress increased serum triglycerides and cholesterol but reduced glucose, whereas heat stress decreased triglycerides [59]. Moreover, low‐temperature stress caused more severe immune dysfunction, evidenced by elevated AKP, AST, ALT, and decreased lysozyme activity [59].

Collectively, temperature is a master regulator of fish gut microbiota, affecting diversity, composition, stability, and function. Short‐term warming often increases diversity but enriches opportunistic pathogens. Long‐term effects depend on warming magnitude; that is, low‐level perturbations allow recovery, whereas high‐level stress drives persistent dysbiosis. Functional consequences include impaired barrier integrity, inflammation, oxidative stress, and altered metabolism. Species‐ and tissue‐specific responses underscore the complexity of host–microbe–environment interactions.

#### 2.3.3 Wild vs. Captive Environments

A growing body of evidence demonstrates that the transition from wild to captive environments profoundly reshapes this microbial ecosystem, though the direction and magnitude of change depend on baseline conditions, diet, water quality, and rearing practices.

Across most species studied, wild fish exhibit higher gut microbial alpha diversity and more complex community structures than their captive counterparts. For instance, wild red‑spotted grouper (*E. akaara*) harbour significantly more diverse gut microbiota than captive individuals, although captive fish display a more intricate co‑occurrence network, enriched in Bacillaceae, Moraxellaceae, and Enterobacteriaceae, while wild fish are dominated by Vibrionaceae, Clostridiaceae, Flavobacteriaceae, and Rhodobacteraceae [68]. Similarly, wild lenok (*B. lenok*) shows enhanced hepatic immune activity (higher lysozyme, catalase, glutathione) and reinforced intestinal morphology compared to farmed fish, which exhibit elevated intestinal stress markers such as malondialdehyde and pro‑inflammatory gene expression [69].

In three cold‑water fish species, *Schizothorax wangchiachii* (herbivorous), *Schizothorax kozlovi* (omnivorous), and *Percocypris pingi* (carnivorous), habitat type outweighs host species in shaping gut microbiota. Wild individuals maintain multiple dominant phyla (Proteobacteria 38%, Fusobacteria 30%, Firmicutes 11%, Cyanobacteria 11%), whereas farmed fish are overwhelmingly dominated by a single phylum (Proteobacteria 82%) [70]. This pattern is mechanistically attributed to the stronger selective filters imposed by captive conditions, which homogenize microbial communities and reduce ecological redundancy [70].

Even when alpha diversity does not drastically decline, captivity simplifies community assembly processes. In Atlantic salmon (*S. salar*), neutral community models applied to wild and farmed populations reveal that most microbes in farmed fish are transient, with no evidence of adaptation to the gut environment [71]. In contrast, wild fish exhibit increasing taxonomic and functional filtering with age, suggesting progressive host selection [71]. Notably, *Mycoplasma* spp. emerged as key adapted residents in both environments, indicating that certain core taxa can persist across habitats [71].

However, exceptions to the general pattern of reduced diversity in captivity exist. In Nile tilapia (*O. niloticus*), samples from an aquaculture centre showed higher alpha diversity than wild samples from Lake Tana, Ethiopia [72]. This counter‑example highlights the importance of baseline environmental conditions; that is, in nutrient‑rich aquaculture systems with diverse feed inputs, microbial diversity may increase, whereas in more homogenised, low‑variation systems, diversity typically declines.

Despite these variations, a core microbiota consistently persists across wild and captive habitats. In red grouper (*Epinephelus morio*), 27 highly abundant ASVs are shared across all sampling years, including *Micrococcus*, *Bacillus*, *Vibrio*, *Pseudomonas*, and *Escherichia*‑*Shigella* [73]. Similarly, cavefish (*Sinocyclocheilus* spp.) share 10 core ASVs, primarily *Aeromonas* and *Cetobacterium*, which are involved in protein digestion and vitamin B12 synthesis [55]. These core taxa likely provide essential functions that are independent of habitat.

Collectively, captivity imposes strong selective filters that generally simplify gut microbial diversity, reduce ecological robustness, and alter functional capacities. While exceptions occur in enriched rearing systems, the overall pattern underscores the need for habitat‑mimicking diets and microbiome‑friendly practices in conservation aquaculture and reintroduction programs.

## 3 Chapter 2. Probiotics for Aquaculture: Not the Ultimate Choice When It Comes to Health Benefits

Almost a century has passed since the concept of probiotics was first developed, and the role of beneficial microbes (later termed probiotics), which play a significant role in promoting and improving health, was demonstrated [74]. Pioneers of probiotic research, such as Stamen Grigorov, Ilya Mechnikov, and Minoru Shirota, championed the beneficial role of lactic acid bacteria and their health‐promoting properties. Industry subsequently adopted this concept and created a multi‐million euro market [75].

Initially aimed at improving human health, the concept of probiotic benefits was later adapted for farm and companion animals [76]. Amid the growing animal health crisis and restrictions on the use of antibiotics to maintain the health of farm animals, probiotics have gained widespread recognition as a sustainable alternative to antibiotics in agricultural practices, including aquaculture. Claimed benefits of probiotics range from improved growth performance to increased disease resistance [77].

However, despite growing interest in probiotics in the academic community and clinical and animal data supporting their health benefits, many studies remain contradictory [78, 79]. In a recently published critical review, the authors attempted to critically evaluate the benefits and limitations of using probiotics in aquaculture. The analysis demonstrates that probiotics, while promising, should not be considered the only universally reliable solution for maintaining the health of aquatic organisms [80]. Here we take a closer look at issues such as strain specificity, environmental variability, host‐microbe interactions, regulatory gaps, and the challenges of translating laboratory results into real‐world production systems.

### 3.1 Strain/Species Specificity and the Problem of Generalization

One of the major issues in probiotic research is the tendency of some investigators to generalize results obtained using different strains of the same microbial species or different microbial species, and/or to extrapolate observations obtained in one eukaryotic organism to other hosts. Probiotic properties are often strictly strain‐specific [81], and, equally important, a given probiotic strain may act in some animal species but not in others, or even act differently in eukaryotic hosts of the same species depending on their age and gender [82].

Environmental conditions in aquatic ecosystems and population composition are just some of the factors that can play a crucial role in the effectiveness of probiotics [83, 84]. Even such a “simple” parameter as temperature requires careful consideration, as most lactic acid bacteria are not fully active at temperatures below 20°C [85, 86]. Therefore, it is difficult to imagine how these specific strains could function as probiotics in aquaculture, where temperatures may fall below 20°C.

Thus, the use of postbiotics offers advantages when developing formulations for use in a variety of environmental conditions. These conditions play a crucial role in the viability and function of probiotics and should be considered as parameters in modeling studies evaluating the use of new beneficial microorganisms (Table 1). Moreover, since postbiotics are bioactive components obtained by inactivating living cells, these environmental conditions may be less important for their biological activity. However, to ensure the biological activity of postbiotics, it is important to verify the physical and biochemical suitability of environmental conditions, as some of the factors listed in Table 1 may still influence their biological effectiveness.

**TABLE 1:** Marine fish and probiotic challenges: Examples.

Fish	GIT	Ecology	Essential microbiota (examples)	Probiotic (examples)
Salmon, e.g., Atlantic salmon (*Salmon salar*), Anadromous life cycle.	Relatively simple, although highly specialized, tubular digestive system, specifically adapted for a carnivorous diet [17, 87]	Fresh water:13°C to 17°C with 12°C to 14°C being optimal. Can tolerate pH 6.5‐8.5, with 6.8‐7.5 being optimal for spawning, egg hatching, juvenile growth, bone and scale development, and transformation into a smolt. Marine water: 8°C to 16°C, with 13°C optimal for growth and feeding; >20°C causes severe stress and can be fatal [88].	Firmicutes and Proteobacteria are the core groups of the GIT microbiota. Lactobacilli, *Leuconostoc*, and *Lactococcus* decrease with aging in the marine environment. *Mycoplasma* is the most abundant in the adult marine stage [89].	*Lpb. plantarum* CCM 8674 and *Limosilactobacillus fermentum* CCM 8675, isolated from the farmed rainbow trout (12°C‐18°C) and incubated in MRS at 37°C, showed some positive effect on salmon's mucosa at approx. 8°C [89], with no information on the microorganisms’ survival and growth in the host (**“postbiotic effect”?**).
Eel, e.g., European eel (*Anguilla anguilla*), American eel (*Anguilla rostrata*), Catadromous life cycle.	Relatively simple and short, optimized for carnivory, water balancing, and fasting [90].	10°C to 20°C for migration and coastal living, with optimal growth and active feeding at 20°C to 25°C. Optimal temperature depends on life stage: juveniles (upstream migration), 12°C to 20°C; active growth (freshwater), 22°C to 28°C [90]. Can tolerate pH 6.5 to 8.0, with optimal being around 7.0 to 7.5.	*Cetobacterium* (some isolates are reported as potential probiotics), *Aeromonas* (reported as dominant in various life stages of farmed eels, contributes to starch digestion), and *Pseudomonas* (found in wild and farmed fish, mucosal health indicator) and Firmicutes. Microbiota varies significantly between species and depends on life stage, environments (wild *vs* farmed), and location in the GIT [91, 92].	*B. subtilis*, *Bacillus licheniformis*, and *Bacillus pumilus* strains are considered promising probiotics for eel, which can effectively suppress *V. anguillarum* and improve fish survival. *Lpb. plantarum* strains are reported to be good immunomodulators [93]. Most of the studied probiotic strains had an optimal incubation temperature higher than the optimal temperature for fish. Interestingly, most reports contain little or no information on the survival and growth of probiotic microorganisms in the host (**“postbiotic effect”?**).
Flathead grey mullet (*Mugil cephalus*), Catadromous life cycle.	A highly specialized gastrointestinal tract designed to process detritus, microalgae, and sediment. It has a thick‐walled stomach and a noticeably long intestine [94].	Optimal temperature is in the range of 20°C to 26°C, but can briefly tolerate temperatures below 20°C or above 28°C [95]. The optimal pH ranges from 7.5 to 8.5.	The bottom‐dwelling, low‐trophic and omnivorous lifestyle of fish are the main factors determining the high diversity of the GIT microbiota [96]. Proteobacteria, Actinobacteriota, and Firmicutes are the most abundant. Seasonal abundance: Chloroflexi (Fall), Spirochaetota and Cyanobacteria (Winter), Bacteroidota (Summer), Staphylococcaceae (Spring). Interesting: High abundance of *Cetobacterium* at 28°C [97]	*Lacticaseibacillus rhamnosus* FS3051 and *Limosilactobacillus reuteri* FS3052 inhibited *Nocardia seriolae* in a scenario where lactobacilli were used as a prophylactic treatment and also exhibited immunomodulatory activity while improving microbiota by enriching *Polynuceobacter*, lactobacilli, and *Enhydrobacter*. *B. subtilis* var. natto NTU‐18 contributed to weight gain, feed efficiency, and specific growth rate [98]. The report contains no information on the survival and growth of probiotic microorganisms in the host (**“postbiotic effect”?**).

### 3.2 Questionable Claims and Regulatory Gaps

The rapid growth of aquaculture has increased interest in probiotics and postbiotics, but it has also led to questionable claims and regulatory gaps that hinder their safe and effective use. The fact that the term “postbiotics” remains undefined by government agencies and international authorities opens the door to speculation and misconduct. One of the main problems is the overgeneralization of probiotic efficacy, with strains touted as possessing a broad range of beneficial properties, despite compelling evidence that probiotic effects are more often strain‐ and host‐dependent. Reviews highlight that many commercial products lack rigorous genomic, in vitro, and in vivo validation, raising concerns about untested safety characteristics, such as the presence of genes coding for various toxicities or antibiotic resistance determinants [99]. Likewise, claims of growth stimulation, immune enhancement, or increased disease resistance are often based on limited or inadequately designed studies, making the reproducibility of results uncertain. Another “scientific conundrum” is that the importance of environmental conditions and host specificity is not considered as determining factors, with some investigators proposing “universal probiotics” that would be a panacea for virtually all eukaryotic organisms and reduce the risk of horizontal transfer of viable‐cell‐associated genetic determinants [100].

Despite the benefits of postbiotics, such as increased stability and reduced risk of horizontal gene transfer, their specific mechanisms of action remain insufficiently characterized. The current literature highlights the lack of standardized definitions, manufacturing protocols, and dose‐response models, which allows manufacturers to market heterogeneous mixtures of metabolites, cell fragments, or supernatants under the same brand name despite significantly different biological activities [101, 102].

Regulatory oversight remains fragmented. Many countries lack specific regulations governing the use of microbial additives in aquaculture, resulting in inconsistent requirements for safety assessment, strain identification, and environmental impact assessment.

Currently, the term “postbiotic” is not officially regulated. In Europe, to the best of the authors' knowledge, ingredients are assessed under general food law or as novel foods (EU Regulation 2015/2283) if the inanimate microorganism lacks a history of safe use, which requires the provision of appropriate toxicological safety data. General and specialized applications of postbiotics, as well as the relevant European regulations associated with these applications that are not specific to postbiotics, have been reviewed in some detail by Vindеrola et al. [103]. In relation to European regulations concerning the use of postbiotics in the food industry, an analysis was carried out using the specific example of bread containing lactic acid bacteria [104]. According to this study, lactic acid bacteria traditionally found in bakery products are categorized as having been used safely before 1997 and are classified as non‐novel foods under EU regulations. Finally, Vinderola et al. [105] investigated the medical use of postbiotics and the regulatory requirements placed on these products in Europe (the reader is referred to Table 2 of the cited article) and stated the absence of any regulations specifically targeting postbiotics.

**TABLE 2:** Biomarkers in postbiotic preparations (examples).

Candidate biomarker	Why is it a biomarker of efficacy/safety/quality	Main methods of determination in the product	Advantages	Limitations	Key sources
Lactate, acetate, propionate, butyrate, and total organic acid profile	Reflect actual fermentation activity; involved in pathogen suppression, pH modulation, epithelial nutrition, gut microbiota modulation; in marine postbiotic products, the SCFA profile is a functional parameter	HPLC‐UV/RID, ion chromatography, GC–MS after derivatization, LC–MS/MS; enzymatic kits can be used for lactate	Inexpensive, rapid, stable, well‐suited for release QC	Low specificity: high acid levels alone do not prove the full bioeffect	[106]
Bacteriocins and bacteriocin‐like peptides: nisin Z, garvicins, weissellicin, etc.	Most specific for antimicrobial claims; directly linked to suppression of fish pathogens, including *Lactococcus garvieae*	Agar diffusion bioassay, RP‐HPLC/UPLC, LC–MS/MS, MALDI‐TOF MS; qPCR of structural genes	High specificity and strong mechanistic relevance	Highly strain‐dependent; often sensitive to proteases and adsorption to matrices	[107, 108, 109, 110]
Cyclic *Bacillus* lipopeptides: surfactin, fengycin, iturin/kurstakin	Associated with membrane damage, antibiofilm effects, and *Vibrio* control; potency markers for Bacillus products	UPLC–MS/MS, HPLC/UPLC‐UV, HPTLC, MALDI‐TOF MS; sample pretreatment with high MeOH or FDSE	High LC–MS sensitivity; direct homolog profile readout	Homolog mixtures complicate standardization; major sorption losses possible without proper sample preparation	[111, 112, 113]
Amicoumacin A	In some anti‐*Vibrio* Bacillus preparations, likely the dominant active compound; a highly specific potency marker	SPE/RP‐HPLC, LC–MS, NMR, activity‐guided fractionation	Very high specificity for anti‐*Vibrio* Bacillus products	Not universal; activity and required levels depend on the target *Vibrio* strain	[114, 115, 116]
Lipoteichoic acids	In *Lpb. plantarum* L‐137, surface LTA expression correlates with IL‐12p40 induction; reasonable identity‐plus‐potency marker for immunomodulatory inactivated Gram+ products	1‐butanol extraction, hydrophobic interaction chromatography, immunoblotting, 2D‐NMR, LC–MS	Strong mechanistic link to immune claims	Low throughput, complex structural variability requires orthogonal purity control	[115, 117] .
Peptidoglycan markers: muramic acid, glucosamine, muramyl/muropeptide signatures	Practical surrogate measure for quantity of inactivated bacterial biomass and innate immune cargo; useful as a universal quantitative marker of “cellular” postbiotics	Acid hydrolysis + HILIC–MS/LC–MS, peptidoglycomics LC–MS	More universal than LTA; good for batch consistency	Less mechanistically specific; hydrolysis simplifies and partly destroys native architecture	[116, 118, 119, 120, 121]
CpG‐rich bacterial DNA and total residual DNA	In fish and shrimp, CpG motifs activate TLR9/TLR21‐like pathways and immune responses; total DNA serves as a marker of preserved inactivated material.	qPCR, ddPCR, PMA‐qPCR to distinguish viable/non‐viable signal; WGS/amplicon sequencing if needed	High analytical specificity and regulatory value	“More DNA” does not mean “better”; no recognized beneficial thresholds	[122, 123, 124]
Exopolysaccharides, including dextran‐like polymers	Linked to mucosal/gut barrier effects, antioxidant and immunomodulatory activity; immune and antiviral effects shown in fish models	Ethanol precipitation, dialysis, SEC‐MALS, HPAEC‐PAD for monosaccharides, FTIR, NMR, GC–MS after hydrolysis	Good identity markers for gut‐health claims	Complex structure–function relationships; both composition and MW profile are needed	[109, 125, 126, 127]
Yeast metabolome: nucleotides/nucleosides, free amino acids, short peptides, organic acids	For yeast SCFP/postbiotics, the most plausible class of growth/gut‐health markers, commercial disclosures are often limited to proximate analysis rather than active metabolites, making multi‐metabolite panels especially important	HILIC‐QTOF‐MS, HILIC‐MS/MS, ^1^H‐NMR, untargeted metabolomics	Suitable for complex yeast matrices and nutritionally active products	Low specificity of individual molecules; of limited value without a reference fingerprint	[128, 129, 130, 131, 132]

Similarly, in the US, the FDA does not have a separate regulatory category for postbiotics, and manufacturers must adhere to general standards for dietary supplements and foods, including GMP and FSMA [11].

Perhaps it is too early to discuss anything that is not already formalized and separately regulated by the main regulatory bodies. Critical reviews note that inappropriate selection, poor formulation stability, and inadequate delivery systems can lead to ineffective or even harmful results, yet these risks are rarely disclosed in commercial claims [10]. Furthermore, long‐term environmental impacts, including changes in microbial communities or unintended selective pressures under the influence of pro‐ and postbiotic products, are poorly understood, leaving regulators without evidence‐based recommendations.

Overall, this field suffers from a lack of harmonization of standards, a limited understanding of mechanisms of action, and marketing practices that outpace scientific evidence [133]. Strengthening the regulatory framework, implementing omics characterization methods, and ensuring transparency in efficacy testing are essential to ensure the responsible use of probiotics and postbiotics in agriculture in general and aquaculture in particular.

### 3.3 Potential Risks and Unintended Consequences

Although probiotics are generally considered safe, their use is not without risks. Potential concerns relate to several aspects and, at some point, may even call into question the significance of the benefits obtained. First, and perhaps most importantly, the use of probiotics or postbiotics should be critically assessed by regulatory authorities, carefully balancing the benefits and risks [134, 135]. Scientific arguments should prioritize economic benefits. Some of the potential risks of probiotic use already identified could be mitigated by postbiotics.

Horizontal gene transfer is a serious concern when using probiotics, as it can facilitate the unintended spread of antimicrobial resistance genes in aquatic environments. Aquatic ecosystems act as major reservoirs of resistance determinants, where mobile genetic elements such as plasmids, transposons, and bacteriophages facilitate gene exchange between coexisting bacteria [136]. Biofilms, which often form in aquaculture systems, further increase this risk by acting as sites for conjugation, transformation, transduction, and even vesicular transfer of DNA, thereby increasing the persistence and mobility of antibiotic resistance genes and other virulence genes [137]. When probiotic strains harbor hidden (latent) or poorly understood resistance determinants, they can contribute to the spread of antibiotic resistance among pathogenic or environmental bacteria, undermining both fish health management and public health overall. Aquaculture data demonstrate that antibiotic resistance genes can easily be transferred to medically important strains, highlighting the need for rigorous genomic screening and regulatory oversight of probiotic candidates [138]; or, in some cases, virulence and antibiotic resistance genes may even be present in probiotics produced commercially for aquaculture [139]. The introduction of probiotic strains into aquaculture systems can lead to ecological disruptions, altering local microbial communities in often unpredictable ways. Research shows that probiotics, as living microorganisms, inevitably alter the structure and abundance of key bacterial groups in the surrounding water and the host‐associated microbiota, affecting heterotrophs, nitrifiers, denitrifiers, and pathogenic or beneficial taxa [140]. Even short‐term probiotic administration can alter microbial diversity, growth rates, and genetic interactions within the resident microbiota, demonstrating that local communities respond rapidly and adaptively to such disturbances [141]. Under larval rearing conditions, changes in the major bacterial microbiota composition have already been documented, including Alteromonadales, Rhodobacterales, and Vibrionales, indicating that introduced strains may interact with or displace natural microbial communities during critical periods of development [142]. Because these ecological responses remain poorly understood, the long‐term consequences of altered microbial networks, such as disruption of nutrient cycling or promotion of opportunistic pathogens, remain a significant scientific and ecological concern. This highlights the need for ecosystem‐level assessments before large‐scale probiotic adoption.

Opportunistic pathogenicity is a serious concern when using probiotics, especially when strains are poorly studied or administered to unprepared patients. Although many probiotic candidates are derived from genera considered by some regulatory agencies as generally safe (such as lactobacilli [143]), some *Bacillus* and *Enterococcus* species possess virulence factors capable of causing disease under favorable conditions, which may challenge the use of probiotics from these taxonomic groups [143, 144, 145].

Insufficient genomic screening can miss traits such as hemolysins, adhesins, biofilm formation, or latent toxin genes, increasing the risk of a strain transitioning from commensal to pathogenic behavior when host defenses are weakened. Fish reared in intensive aquaculture conditions are particularly vulnerable to such opportunistic infections due to stress, overcrowding, suboptimal water quality, and the use of stimulants [146]. Case reports from aquaculture [147] and human [148] studies have shown that even traditionally “safe” strains can cause bacteremia or systemic infection when host immunity is compromised and/or probiotic supplementation is discontinued. These risks highlight the need for careful strain identification, whole‐genome sequencing, and standardized virulence assessment before probiotics are approved for commercial use.

Disruption of the healthy microbiome can be a serious problem when using probiotics [23]. This problem can be addressed to some extent with postbiotics, especially early in life, when host‐microbe interactions shape long‐term physiology. The formation of a stable, diverse microbiome is a tightly regulated ecological and immunological process influenced by host genetics, environmental exposure, and microbial successional dynamics [149]. Introduction of exogenous probiotic strains during these critical periods may disrupt this trajectory by altering microbial succession, suppressing key taxa, or accelerating colonization patterns in ways that differ from natural development [150, 151]. Such disruptions may have long‐term consequences because early microbial community formation influences nutrient metabolism, immune maturation, epithelial barrier function, and disease susceptibility later in life. Data from vertebrates show that early‐life microbiome perturbations can lead to persistent changes in microbial community composition and host phenotype [152]. These findings highlight the need for stage‐specific evaluation of probiotics and caution against routine, untargeted supplementation during sensitive developmental periods.

## 4 Chapter 3. Postbiotics: An Alternative to Probiotics, With Promises and Challenges

### 4.1 Postbiotics: Determinadum Est

For a long time, the term “postbiotics” has been used in various ways: some authors have referred to metabolites and cell‐free fractions, while others have included inactivated microbial biomass and cellular components. Such diversity creates difficulties in comparing and replicating research findings [11]. In 2021, the International Scientific Association for Probiotics and Prebiotics (ISAPP) adopted a definition of postbiotics, specifying that they are preparations of inactivated microorganisms and their components with proven benefits for the host. Such “clarification of terminology” is important for aquaculture because the viability of probiotics is reduced when feed production processes, such as high temperatures or pressure during pelletization, are used. Furthermore, the open aquatic environment places greater demands on biosafety and the standardization of microbial interventions.

#### 4.1.1 Terminological Inconsistencies and Their Origins

The Latin term determinadum est (“to be determined”) aptly reflects the situation with postbiotics: it gained popularity before clear boundaries for their definition were established. There are at least two main interpretations in the literature: (i) “postbiotics” as cell‐free metabolic products (e.g., cell‐free supernatant) and (ii) “postbiotics” as preparations made from inactivated microbial cells or their components, with the possible presence of metabolites. In practice, this means that the same term can refer to interventions that differ in composition and mechanism of action, leading to different dosages, risks, and quality control requirements [153].

Historically, early and frequently cited reviews placed particular emphasis on “soluble factors” and metabolites. For instance, Aguilar‐Toalá et al. [154] defined postbiotics as soluble products or metabolic by‐products that are secreted by living bacteria or released upon cell lysis, citing examples such as organic acids, teichoic acids, and peptidoglycan‐derived molecules [154].

No clear distinction has been established between “cell‐free metabolites” and “inactivated cells or cell fragments,” which has contributed to a blurring of the terminology; Żółkiewicz et al. [155] explicitly defined postbiotics as “any substance” released or produced by a microorganism that benefits the host. Reviews focusing on the relationship between “postbiotics and paraprobiotics” noted that this field is conceptually heterogeneous and requires standardization of terminology and methods for evaluating preparations [156].

#### 4.1.2 What Constitutes a Postbiotic in Aquaculture and Why We Vote for the ISAPP 2021 Definition

The concept of the term “paraprobiotic” developed in parallel: Taverniti and Guglielmetti [157] proposed this term on the basis that the word “probiotic” should be restricted to live microorganisms. They used the term “paraprobiotic” to refer to inactivated cells or cell fractions capable of conferring benefits [157]. Subsequent reviews have systematized the methods of inactivation and the range of biological effects of “inactivated probiotics,” which once again highlighted the need to establish a unified terminological framework [158].

The 2021 ISAPP Consensus defines a postbiotic as “a preparation containing inactivated (non‐viable) microorganisms and/or their components that confers a health benefit to the host” [11]. An important clarification is that postbiotics are specifically inactivated microbial cells (with or without metabolites); purified metabolites and vaccines are not considered postbiotics. Indications and benefits must be substantiated for a specific application [159]. The FAQ publication clarifies contentious issues relating to applicability, the term “inanimate,” safety expectations, as well as questions of quantification and the evidence base for “health benefits” [153].

The ISAPP definition is particularly appropriate for aquaculture because it (a) links the term to a specific type of preparation (e.g., encapsulated microbial biomass or components), (b) requires a demonstration of efficacy in the target form or stage, and (c) promotes reproducible standardization of the production process and dosage. This approach directly reflects real‐world aquaculture conditions: for example, a study on *Penaeus vannamei* (white shrimp) notes that high temperature and pressure during feed pelletization can reduce the activity of probiotics or cause their degradation, prompting a comparison of live and inactivated forms [160].

#### 4.1.3 Components Of Postbiotic Preparations and Their Significance in Aquaculture

In practice, it is useful to group postbiotic preparations by their main components, bearing in mind that actual products are often mixtures. Metabolites (as auxiliary components of the preparation, rather than “purified metabolites”): substances such as organic acids, antimicrobial peptides/bacteriocins, and others may help to suppress pathogens and maintain the gut environment. However, ISAPP emphasizes that purified metabolites should not be labeled as standalone postbiotics [133].

**Cellular components**: Elements of the cell wall and surface structures (e.g., teichoic acids, peptidoglycan‐derived molecules, surface proteins, and exopolysaccharides) are often regarded as key molecular “signals” that modulate innate immunity and mucosal barrier functions. Such components are naturally present in preparations of inactivated cells and cell lysates [161].

**Lysates and cell fragments**: Mechanical or physical disruption (or partial lysis) produces a mixture of cell wall fragments, proteins, nucleic acids, and soluble factors. In aquaculture, this can be technologically advantageous due to its storage stability and compatibility with feed production processes; studies of inactivated strains and products in feed indicate that this approach is suitable for fish models (e.g., the use of functional ingredients from heat‐killed *Lactiplantibacillus plantarum* in experiments with salmonids [162].

**Extracellular vesicles (bEVs) and vesicular cargo**: A review of bEVs explicitly refers to them as “emerging postbiotics”, highlighting their potential biological effects and the need for in‐depth study, production control, and safety assessment. For aquaculture, this is a promising but methodologically more complex class (particularly in terms of analytics and standardization) [163].

#### 4.1.4 Bacteriocins and Other Microbial Inhibitory Metabolites: Mechanistic Links Between Probiotics and Postbiotics

Although probiotics and postbiotics are often presented as distinct microbial intervention strategies, they are closely interconnected at a mechanistic level. Many beneficial effects attributed to probiotic microorganisms are mediated not solely by microbial viability or colonisation, but through the production of bioactive molecules that influence microbial communities and host responses. These microbial products include antimicrobial peptides, bacteriocins, organic acids, enzymes, siderophores, extracellular polysaccharides, and immunomodulatory cellular components. In this context, postbiotics can be viewed not as replacements for probiotics, but as strategies designed to preserve and deliver selected beneficial microbial functions independently of the continued viability of the producing organism.

Bacteriocins represent one of the best‐characterised examples of this probiotic–postbiotic continuum. These ribosomally synthesised antimicrobial peptides are produced by diverse bacterial taxa and contribute to microbial competition, ecological fitness and niche establishment [164, 165, 166]. Although initially regarded primarily as natural antibiotics, bacteriocins are increasingly recognized as ecological mediators capable of shaping microbial communities through highly selective interactions. Consequently, the identification of bacteriocin‐producing strains has historically represented a functional strategy for probiotic discovery, even before the emergence of the postbiotic concept.

The development of bacteriocin‐producing probiotics illustrates this overlap between living microorganisms and their bioactive products. The oral commensal *Streptococcus salivarius* K12 provides one example, having originally been selected because of its production of bacteriocin‐like inhibitory substances (BLIS) active against selected pathogenic streptococci. Subsequent studies identified these inhibitory activities as lantibiotic peptides, including salivaricin A2 and salivaricin B [167]. Although administered as a viable probiotic, many ecological activities of K12 are mediated through defined extracellular antimicrobial molecules and associated regulatory interactions, demonstrating that probiotic efficacy may depend substantially on molecules that are themselves postbiotic‐like functional components.

Similar principles apply within aquatic microbial ecosystems, where microbial metabolites may influence pathogen competition, microbial balance, and host health. Aquaculture environments contain complex microbial communities in which ecological interactions between microorganisms can determine whether pathogenic species successfully establish. Inhibitory metabolites produced by aquatic bacteria, including bacteriocin‐like compounds and siderophores, therefore represent potential mechanisms through which beneficial microorganisms suppress pathogens.

An early demonstration of this principle was provided by Pybus et al. [168], who showed that growth of the salmon pathogen *Vibrio ordalii* was inhibited by a siderophore produced by *Vibrio anguillarum* strain VL4355. This inhibition resulted from microbial competition for iron availability, illustrating how naturally produced bacterial metabolites can regulate pathogen growth through mechanisms other than direct antimicrobial killing. Such observations anticipated current concepts in postbiotic development, where defined microbial functions rather than simply microbial presence may be exploited to improve host health. This represented one of the earliest demonstrations in aquaculture that microbial iron‐chelating metabolites could selectively inhibit fish pathogens [169]. Furthermore, because iron availability regulates expression of numerous bacterial virulence determinants, siderophore‐mediated modification of iron accessibility may influence not only pathogen proliferation but also pathogen behavior and virulence expression.

In addition to direct antagonistic activity, microbial metabolites may contribute to aquaculture health through modulation of host immunity, enhancement of barrier function and alteration of microbial signalling networks. These broader ecological effects reinforce the concept that beneficial microorganisms often act through integrated mechanisms involving both the organism and its metabolic products [80].

However, application of bacteriocins, siderophores and other inhibitory metabolites as postbiotic strategies requires careful evaluation. Their effectiveness may depend on molecular stability, concentration, delivery method, environmental conditions, and specificity toward target organisms. Therefore, as with probiotics, successful postbiotic development requires mechanism‐based selection and validation within the intended host and ecological context.

Recognition of probiotics and postbiotics as a functional continuum provides a useful framework for future aquaculture applications. Probiotic research has identified microorganisms capable of generating beneficial biological activities, whereas postbiotic approaches offer opportunities to deliver selected microbial functions in stable, controlled, and potentially safer formats.

#### 4.1.5 Postbiotics as Anti‐virulence Agents: Suppressing Pathogenic Behavior Rather Than Microbial Survival

The traditional approach to controlling bacterial diseases in aquaculture has focused largely on the elimination or growth suppression of pathogenic microorganisms. However, many important aquatic pathogens are not obligate pathogens, but rather opportunistic members of aquatic microbial communities whose disease potential depends on the expression of specific virulence traits. Species such as *Vibrio harveyi*, *Vibrio parahaemolyticus*, and *Aeromonas hydrophila* can persist within aquatic ecosystems and host‐associated microbiotas, with pathogenicity emerging under favourable environmental conditions such as host stress, microbial imbalance, increased temperature, or high stocking density. This ecological context provides opportunities for alternative disease‐control strategies based on attenuation of virulence rather than direct bacterial killing [8, 86, 101].

An emerging concept in postbiotic research is that microbial‐derived molecules may function as anti‐virulence agents, interfering with the regulatory systems that pathogens require for successful colonisation and disease development. Unlike classical antimicrobial compounds, anti‐virulence molecules do not necessarily inhibit bacterial growth, but instead reduce the ability of pathogens to damage the host or dominate microbial communities. Such approaches may impose weaker selective pressure for resistance development while helping maintain the balance of resident microbial ecosystems [80, 86, 101].

A major target of anti‐virulence activity is *quorum sensing* (QS), the cell‐density‐dependent communication system used by many aquatic pathogens to coordinate collective behaviors. In *Vibrio* spp. and *Aeromonas* spp., QS regulates multiple virulence‐associated functions, including biofilm formation, motility, secretion systems, extracellular enzyme production, and toxin expression. Microbial metabolites present in postbiotic preparations may interfere with these signalling networks by degrading signalling molecules, blocking signal recognition, or modifying downstream regulatory pathways. Such *quorum quenching* activities have the potential to reduce pathogen fitness within the host environment without causing broad disruption of the microbiota [1, 86, 101].

Biofilm formation represents another important target for postbiotic intervention. Biofilms contribute substantially to the persistence of aquatic pathogens by enhancing survival under environmental stress conditions and increasing tolerance to antimicrobial treatments and host defence mechanisms [170]. Postbiotic‐associated molecules, including organic acids, biosurfactants, bacteriocin‐like compounds, extracellular enzymes, extracellular vesicle‐associated factors and other microbial metabolites, may interfere with bacterial attachment, extracellular matrix formation and biofilm maturation. Suppression of biofilm development may be particularly valuable in hatchery systems, larval production facilities and recirculating aquaculture systems, where surface‐associated microbial communities strongly influence pathogen persistence and disease risk [1, 101].

Experimental studies support the feasibility of targeting virulence regulation rather than microbial survival. For example, quorum‐quenching bacteria and their extracellular products have been shown to suppress *A. hydrophila* virulence‐associated characteristics, including biofilm formation, extracellular enzyme activity, and haemolytic activity, thereby enhancing host protection without relying primarily on growth inhibition [171]. Similarly, metabolites produced by candidate probiotic bacteria such as *Bacillus velezensis* have been shown to interfere with QS and biofilm formation by aquatic pathogens, including *V. anguillarum*, further supporting the potential role of microbial products as postbiotic‐associated anti‐virulence agents [172].

Beyond QS and biofilm inhibition, postbiotic components may influence additional virulence‐associated phenotypes, including bacterial adhesion, invasion, motility, secretion of hydrolytic enzymes, and toxin production. Suppression of these functions may shift potentially pathogenic microorganisms toward a less damaging ecological state rather than eliminating them. Such a strategy is consistent with modern microbiome management approaches, where preservation of a stable microbial equilibrium may be preferable to repeated attempts at broad‐spectrum pathogen removal [86].

Recognition of anti‐virulence activity expands the potential role of postbiotics beyond direct antimicrobial effects and host immunomodulation. Future development of aquaculture postbiotics may therefore involve screening candidate microorganisms not only for pathogen inhibition or immune stimulation, but also for their ability to interfere with microbial communication, virulence regulation and pathogen behavior. This approach aligns with the emerging concept of precision postbiotics, in which specific microbial functions are selected and delivered according to host species, pathogen challenges, and environmental conditions [101].

### 4.2 Implications of Terminological Ambiguity for Methodology, Experiments, and Regulation

Ambiguous definitions lead to three types of issues. The primary distinction is methodological: probiotics are quantified by viability (CFU), whereas postbiotics require different metrics (e.g., biomass, “cell equivalents,” integrity markers, or molecular indicators), along with accurate reporting of strains and inactivation parameters. The ISAPP 2021 agreement indicates that various analytical methods (e.g., cytometry, qPCR, etc.) may be beneficial for evaluating biomass or preparations; nonetheless, there is no definitive “CFU equivalent” for postbiotics [11]. The associated “quantification perspective” [161] underscores the absence of a universal “gold standard.” It suggests methodologies for selecting suitable techniques and correlates them with regulatory standards and labeling obligations, such as those required for ensuring compliance in various industries like pharmaceuticals and food safety [161].

The second is experimental: if “postbiotic” in one study signifies a cell‐free supernatant, while in another it indicates heat‐killed biomass, then both the hypotheses and the control parameters vary (e.g., matrix/carrier controls, comparisons between “live” and “inactivated” forms, and, where applicable, fractionation into cellular and metabolic components). In aquaculture, this distinction is particularly important because effects depend on species, age/developmental stage, feed, etc., as shown by reviews of the use of heat‐inactivated formulations and postbiotics as feed additives [173, 174].

The third aspect is regulatory and commercial: the lack of a defined nomenclature and uniform guidelines for product description hinders the evaluation of quality, safety, and the validation of benefit claims. An industry review distinctly defines the postbiotics market as fragmented, necessitating enhanced categorization and terminology for regulatory and commercial frameworks [133].

This review defines a postbiotic, in accordance with the ISAPP definition, as a preparation of inactivated microorganisms and/or their components that, when supplied at an acceptable dosage, provides a verified health benefit to the host [11].

To categorize data as “postbiotic” in aquaculture contexts, we deem the following criteria indispensable: (a) Identification and characterization of the progenitor strain(s) prior to inactivation; (b) Description of the inactivation method, with verification of non‐viability or acceptable residual viability levels, if applicable; (c) Use of a reproducible dose metric (not CFU, but biomass/cell equivalents/validated markers); (d) Inclusion of appropriate controls to distinguish effects attributable to cellular components from those of the metabolic fraction; (e) Definition of aquaculture‐relevant endpoints (growth/feed conversion, mucosal barrier and innate immunity indicators, stress resilience, and infection challenge models), considering feeding technology factors (e.g., pelleting) that may effectively convert a probiotic into an inactivated form [133].

### 4.3 Are There More Pros Than Cons? Compared to Probiotics, are Postbiotics More Predictable/Controllable During Preparation and Storage, Safer, etc.?

Probiotics have been widely applied in modern aquaculture as functional biomodulators that improve growth performance, immune responsiveness, disease resistance, and gut health in fish and crustaceans [175]. However, their efficacy depends on the presence of viable cells and, in many cases, on their ability to survive feed processing, storage, and passage through the gastrointestinal tract. This creates technological and biological uncertainty because live probiotic cultures are sensitive to extrusion or pelleting temperatures, prolonged storage, oxygen and moisture exposure, and environmental fluctuations typical of aquaculture systems [10, 176, 177]. These constraints have stimulated interest in postbiotics, defined as preparations of inanimate microorganisms and/or their components that confer a health benefit on the host, as a more controllable alternative to live probiotics [11, 177].

### 4.4 Comparative Advantages of Postbiotics

#### 4.4.1 Technological Robustness and Compatibility With Feed Production

The most evident advantage of postbiotics is their higher compatibility with industrial aquafeed production. Unlike probiotics, postbiotics do not require the preservation of viable cells during processing; therefore, they are less vulnerable to the high temperatures used during feed extrusion, pelleting, drying, and storage. This property allows postbiotic preparations to be incorporated into feeds without the strict viability‐protection strategies that are often necessary for live probiotic cultures [10, 173]. In practical terms, this improves reproducibility during manufacturing and can reduce costs associated with cold‐chain logistics, specialized packaging, and short shelf life [178].

Thermal or chemical inactivation may also preserve functionally relevant microbial structures and metabolites. For example, heat‐inactivated preparations of *Lpb. plantarum* have been reported to retain antibacterial and antifungal activity, indicating that microbial bioactivity can persist even after cell viability is lost [1, 173]. Thus, postbiotics are not simply “inactivated microbial preparations”; rather, they represent standardized preparations in which cell‐wall fragments, secreted metabolites, enzymes, organic acids, extracellular vesicles, and other bioactive molecules may remain active after inactivation.

#### 4.4.2 Greater Predictability and Easier Standardization

Postbiotics offer greater predictability because their biological activity does not depend on survival, multiplication, or colonization by living microorganisms. The final composition of a postbiotic product is defined during production through the choice of producer strain, nutrient medium, cultivation conditions, growth phase, inactivation method, and formulation strategy. When these parameters are controlled, the resulting product can show more consistent composition and more reproducible biological effects than live probiotics, whose performance depends on host species, resident microbiota, water quality, environment, and stress status [1, 178].

This distinction is particularly important in aquaculture, where probiotic responses are often inconsistent across species and production systems. Live probiotics must remain viable in the feed and then interact with a highly variable intestinal ecosystem. Postbiotics, by contrast, act through preformed molecules and microbial structures, making their effects less dependent on colonization success. Accordingly, postbiotic preparations have been associated with improvements in growth, survival, intestinal health, immune parameters, and stress tolerance in aquatic animals, including hybrid grouper exposed to ammonia stress after supplementation with preparations derived from *Lactobacillus acidophilus* and *Saccharomyces cerevisiae* [179].

#### 4.4.3 Improved Safety Profile

The absence of viable cells gives postbiotics a more favorable safety profile than probiotics. Live probiotic microorganisms, even when generally recognized as safe, may under unfavorable conditions present risks associated with intestinal translocation, uncontrolled persistence, opportunistic infection, disturbance of the native microbiota, and dissemination of virulence or antimicrobial‐resistance determinants [11, 155, 176]. In intensive aquaculture, these concerns are amplified by high stocking density, fluctuating temperature, deteriorating water quality, and stress‐induced immunosuppression [86].

A central advantage of postbiotics is that they remove risks directly linked to microbial viability. They cannot proliferate in the host or aquatic environment, cannot revert to virulence, and greatly reduce risks associated with replication, persistence, and viable‐cell‐mediated transfer of antimicrobial resistance determinants as living cells can. This is especially relevant because aquaculture‐associated bacteria, including *Vibrio alginolyticus* and other marine or gut‐associated microorganisms, can function as reservoirs and vectors of antimicrobial‐resistance genes [68, 180]. Commercial probiotic products have also been reported to contain antimicrobial‐resistance determinants, including *tet*M, *erm*B, *sul*1, and *dfr*G, raising concerns about their transfer to the resident microbiota [68]. Postbiotic use therefore mitigates, although does not automatically replace, the need for quality control and the risks associated with viable probiotic cells carrying resistance genes or virulence factors [10, 101].

The safety advantage is also illustrated by strain‐specific examples. Domínguez‐Maqueda et al. [181] noted that the live strain *Shewanella putrefaciens* Pdp11 may raise concerns about unpredictable reversion to virulence or the acquisition of antibiotic resistance, whereas its extracellular products may provide antimicrobial or immunomodulatory activity without introducing viable cells into the host or environment [181]. Similarly, clinical and veterinary literature has documented that some probiotic microorganisms can, under specific circumstances, contribute to dysbiosis, mucosal‐barrier disruption, or severe systemic infection; postbiotics substantially reduce these viability‐dependent hazards [11].

#### 4.4.4 Functional Efficacy Under Variable Aquaculture Conditions

Because postbiotic activity is mediated by preformed structural components and metabolites, it is generally less affected by external environmental factors than the activity of living probiotics. It has been previously discussed how aquatic environments, stocking density, and stress can strongly influence host physiology, probiotic survival, and metabolic activity, while the activity of inanimate preparations is less affected by these factors [1, 101, 177, 182]. Nevertheless, this advantage should not be overstated: extreme temperature, oxidative stress, pH shifts, or enzymatic degradation can still reduce the activity of specific postbiotic components.

### 4.5 Limitations and Unresolved Problems of Postbiotic Application

#### 4.5.1 Composition Depends Strongly on Production Conditions

Controllability of postbiotics is also their main technological challenge. Although their composition can be standardized, it is not inherently fixed: it depends on the producer strain, fermentation conditions, growth stage at harvest, inactivation protocol, and subsequent formulation [1, 155]. Consequently, different strain‐environment‐inactivation combinations may yield preparations with distinct metabolite profiles, hydrolytic activities, antibacterial, antiviral, and immunomodulatory properties.

Several studies support this point. García‐Márquez et al. [96] showed that optimizing growth conditions can improve the uniformity and biological characteristics of postbiotic preparations, supporting their suitability for industrial manufacturing. Domínguez‐Maqueda et al. [183] reported that extracellular products of *S. putrefaciens* Pdp11 exhibit hydrolytic, antibacterial, and antiviral activities that depend on medium composition, temperature, growth phase, and salinity. Similarly, *Lpb. plantarum* postbiotics may retain antimicrobial activity after inactivation, but the magnitude and spectrum of this activity vary depending on cultivation and inactivation conditions [184]. Thus, each producer strain and process configuration requires separate validation.

#### 4.5.2 Differential Stability of Postbiotic Components

Although postbiotics are generally more stable than probiotics, their components do not have identical stability. Proteinaceous molecules, such as bacteriocins and enzymes, as well as nucleic acids, may be degraded by proteases and nucleases, thereby reducing biological functionality [154]. Organic acids, including lactic acid, acetic acid, and short‐chain fatty acids, are not necessarily degraded completely, but their functional impact can decline through dissociation, dilution in the aquatic environment, or utilization by resident microorganisms. Extracellular bacterial vesicles are another promising postbiotic fraction, yet they can be damaged by lipid oxidation and membrane integrity disruption under physicochemical stress [185].

In contrast, exopolysaccharides are usually more stable, although they may undergo enzymatic hydrolysis. Cell wall‐associated structures, such as peptidoglycan, lipopolysaccharides, and teichoic acids, tend to preserve structural integrity and immunomodulatory potential for longer periods [156]. Therefore, formulation design must consider the differential stability of each bioactive fraction. Protective technologies, including microencapsulation and optimized storage conditions, remain important even for postbiotic products.

#### 4.5.3 Limited In Vivo Evidence, Dosing Uncertainty, and Strain Specificity

A second major limitation is that the evidence base for postbiotics in aquaculture remains less extensive than that for probiotics. Many studies are still limited to specific host species, short experimental periods, narrow environmental conditions, or a small number of producer strains. Long‐term effects, optimal dosing regimens, interactions with feed ingredients, effects on different life stages, and performance under commercial farm conditions require further investigation [176].

Postbiotic effects are also highly strain‐specific. Kumar et al. [186] demonstrated that postbiotics derived from *Bacillus subtilis* showed stronger biological activity than those obtained from the closely related *Bacillus amyloliquefaciens*. This indicates that the efficacy of one postbiotic preparation cannot be extrapolated to another, even when producer strains belong to related taxa. For practical implementation, this considerably increases the number of validation studies required before commercial use.

#### 4.5.4 Incomplete Understanding of Molecular Mechanisms

The molecular mechanisms of postbiotic action are still incompletely characterized. General immunological pathways have been proposed, particularly those involving pattern‐recognition receptors such as Toll‐like receptors and NOD‐like receptors; however, the precise molecular targets and downstream signaling events remain unknown for many postbiotic preparations [155]. Recent work has begun to fill this gap. For example, Liu et al. [187] reported that paraprobiotics and postbiotics derived from *Bacillus siamensis* LF4 modulate immunity through TLR2/TLR5‐MyD88 signaling, thereby regulating the p38 MAPK and NF‐κB pathways. Jafarzadeh et al. [188] likewise confirmed the involvement of PRR‐mediated mechanisms in the effects of paraprobiotics and postbiotics during advanced calciferosis. Nevertheless, for most producer strains and postbiotic fractions, the relevant molecular targets remain insufficiently defined, limiting rational formulation design and precision feeding strategies (Table 2).

Overall, postbiotics appear to offer more practical advantages than disadvantages compared with probiotics in aquaculture, particularly for industrial feed production. Their strongest advantages include technological stability, independence from viable‐cell survival, improved safety, better manufacturing controllability, and lower sensitivity to environmental variability (Table 3).

**TABLE 3:** Comparative assessment of postbiotics and probiotics for aquaculture.

Criterion	Main advantage of postbiotics	Remaining limitation	Key sources
**Feed processing and storage**	Do not require viable cells; better tolerance to extrusion, pelleting, drying, and storage.	Some metabolites and vesicles can still lose activity under extreme stress or poor storage.	[10, 189]
**Predictability**	Effects are less dependent on colonization and survival in the host gut.	Composition still depends on strain, medium, growth phase, and inactivation method.	[1, 178, 183]
**Safety**	No proliferation, translocation, reversion to virulence, or transfer of resistance genes by viable cells.	Quality control is still required to exclude harmful residual compounds or inconsistent formulations.	[10, 11, 65, 68, 101]
**Biological efficacy**	Can improve growth, immunity, survival, gut health, and stress resistance.	Efficacy is strain‐ and host‐specific; optimal dosing is not fully established.	[176, 179, 186]
**Mechanistic basis**	Some pathways involving PRRs, TLRs, MyD88, MAPK, and NF‐κB have been identified.	Mechanisms remain unclear for many postbiotic fractions and producer strains.	[134, 155, 188]

These properties make them attractive for large‐scale aquaculture applications, as well as for production systems where rearing parameters vary over time to meet the fish's natural requirements. At the same time, postbiotics should not be considered a universally superior replacement for probiotics. Their efficacy depends on the producer strain, cultivation conditions, inactivation method, and the stability of individual bioactive components. Moreover, mechanistic data, long‐term in vivo trials, dose‐response studies, and standardized quality‐control protocols remain insufficient for many preparations. Therefore, the most scientifically defensible conclusion is that postbiotics are a safer, more stable, and more manageable alternative to probiotics, but their successful application requires strain‐specific validation, standardized manufacturing, and clear characterization of active components (Table 4).

**TABLE 4:** Pros and cons of using probiotics and postbiotics for aquatic animals.

Challenges	Biologically active formulation		Notes
	Probiotic	Postbiotic	
Survival and performance under various environmental factors	Influenced by temperature, pH, salinity of the environment, etc.	No need to “survive”; stability and performance can be improved through intelligent formulation	Postbiotics may provide more predictable and consistent performance
Ability/need to multiple	YES	NO	Higher probability of positive results from postbiotics
Genome variability	Can present challenges, particularly in the case of lactobacilli, such as inconsistency in performance and transfer of pathogenic factors	There are no such issues; moreover, molecular characterization of the microorganism used for the production of the postbiotic formulation and determination of the formulation's chemical composition are mandatory	When used in aquatic animals, postbiotics may be safer and more reliable in providing the expected effect than probiotics
Over‐growth or insufficient growth	Negative effects on the host organism in case of excessive growth or decreased effectiveness in case of insufficient growth	No such issues	When used in aquatic animals, postbiotics may be safer and more reliable in providing the expected effect than probiotics
Quality and safety	Challenges in controlling the number of live microorganisms in formulations and in the host organism	No such issues	No such issues for postbiotics; moreover, the inanimate microorganism must be molecularly characterized, and the chemical composition of the formulation must be identified
The eukaryotic host's anatomy and physiology	Can present a challenge; not every probiotic will have the same effect on different aquatic organisms. Probiotics can influence the host microbiota, reducing its diversity (this has been demonstrated in poultry)	Most likely, not an issue; any challenges can be overcome with intelligent design of the delivery vehicle	Postbiotics may have more predictable and reproducible beneficial effects on aquatic hosts than probiotics
Impact on the environment	Possible impacts, both positive and negative	Negative effects are unlikely; postbiotics may increase the diversity of the host microbiota and ecosystem	While probiotics may improve the microbial ecology of a water reservoir, postbiotics are likely to be safer and more controllable
Aquatic animal open systems’ challenges	The ability of the probiotic to colonize the host's ecological niche will be challenged by the constant flow of water	Controlled delivery of postbiotics using intelligently designed vehicles can positively impact their efficacy in aquatic organisms	Postbiotics may have more predictable and reproducible beneficial effects on the aquatic host
Expected benefits	Depends on multiple variable factors	More predictable if challenges are identified and addressed appropriately	Postbiotics may have more predictable and reproducible beneficial effects on the aquatic host

### 4.6 Host Physiological and Immunometabolic Biomarkers Associated With Postbiotic Application in Aquaculture

One of the important approaches to assessing the efficacy of postbiotics is the analysis of biomarkers—objective measurable indicators that reflect biological and pathological processes or the organism's response to exposure to various factors. The use of biomarkers enables a comprehensive evaluation of the beneficial effects of postbiotics on the organisms of hydrobionts, including the reduction of inflammatory processes, normalization of metabolism, and enhancement of the immune system, making them an effective tool for the sustainable development of aquaculture [190].

Biomarkers are widely used in clinical biochemistry and molecular biology to monitor health status and to assess the effectiveness of biotechnological interventions, including postbiotics. In the context of aquaculture, the following groups of biomarkers are of particular importance: (A) Inflammatory markers, such as C‐reactive protein (CRP), interleukin‐6 (IL‐6), and tumor necrosis factor‐alpha (TNF‐α), allow the level of inflammatory processes in the organism to be assessed. A decrease in their concentration under the influence of postbiotics indicates an anti‐inflammatory effect. (B) Metabolic biomarkers, including indicators of glucose levels, lipid metabolism, and enzymatic activity, reflect the state of metabolism and the organism's energy balance. Their normalization indicates improved physiological status and adaptive capacity. (C) Immunological biomarkers, such as immunoglobulin levels, leukocyte activity, and cytokines, characterize the state of the immune system. Changes in these indicators under the influence of postbiotics indicate enhanced immune protection and increased resistance to infections [191].

#### 4.6.1 Inflammatory Biomarkers

Inflammatory biomarkers are biological indicators that objectively assess the intensity, duration, and systemic effects of the immune‐inflammatory response in the organism. They are molecular indicators of immune system activation and reflect the response to infection, tissue damage, and stress exposure. The most important inflammatory biomarkers include C‐reactive protein (CRP), interleukin‐6 (IL‐6), and tumor necrosis factor‐alpha (TNF‐α) [192].

##### 4.6.1.1 CRP (C‐reactive protein)

C‐reactive protein is an acute‐phase protein synthesized in the liver under the influence of IL‐6. It is considered a rapid and sensitive marker of inflammatory processes. Elevated CRP levels are observed in infectious diseases, tissue necrosis, and systemic inflammation. CRP is widely used in clinical and experimental studies to assess the intensity of inflammation [193].

##### 4.6.1.2 IL‐6 (Interleukin‐6)

Interleukin‐6 is a multifunctional cytokine that plays a central role in the initiation and regulation of inflammatory reactions. IL‐6 is produced by macrophages, endothelial cells, and T lymphocytes. It stimulates the synthesis of acute‐phase proteins and enhances the systemic immune response. Elevated IL‐6 levels are associated with chronic inflammation and infectious diseases [194].

##### 4.6.1.3 TNF‐α (Tumor necrosis factor‐alpha)

TNF‐α is a key pro‐inflammatory cytokine involved in immune cell activation, regulation of apoptosis, and initiation of the inflammatory cascade. It is secreted by macrophages and stimulates the production of other cytokines, including IL‐1β and IL‐6. Elevated TNF‐α levels are associated with various inflammatory and infectious pathologies [192].

In aquaculture, biomarkers such as CRP, IL‐6, and TNF‐α are important for assessing the immune status of aquatic organisms. The application of postbiotics, probiotics, and functional feeds reduces the levels of these biomarkers, indicating attenuation of inflammatory processes and stabilization of the immune system. At the same time, these biomarkers are highly sensitive to environmental factors such as water temperature, oxygen levels, and stress exposure [195]. Inflammatory biomarkers, including CRP, IL‐6, and TNF‐α, are key molecular indicators for assessing immune system activity. They enable objective determination of the level of inflammation, the intensity of the immune response, and the organism's ability to resist pathogens. In aquaculture systems, these biomarkers are widely used to evaluate the efficacy of postbiotics and functional feeds.

#### 4.6.2 Immunological Indicators and Biomarkers

Immunological biomarkers are a principal tool for the objective assessment of the immune system status of aquatic organisms. Changes in these indicators reflect the nature of the immune response and the degree of organismal resistance to pathogenic agents. In particular, a decrease in pro‐inflammatory cytokine levels accompanied by an increase in protective factors under the influence of postbiotics confirms their pronounced immunomodulatory effect [186]. In assessing immunomodulatory processes, immunological biomarkers are regarded as a key scientific tool. These include pro‐inflammatory cytokines (IL‐1β, IL‐6, TNF‐α, IFN‐γ, IL‐12), complement system activity, lysozyme levels, immunoglobulin IgM content, and the phagocytic index. These indicators allow the activity of the immune system, the level of oxidative stress, and the overall physiological state of aquatic organisms to be objectively evaluated.

The application of postbiotics in industrial aquaculture is of considerable importance for immune system regulation. It has been established that they activate innate immune mechanisms, enhance phagocytosis, and regulate cytokine production. However, their efficacy may depend on environmental factors such as water temperature, dissolved oxygen level, stocking density, and feed quality. In particular, tumor necrosis factor‐alpha (TNF‐α) and interleukin‐6 (IL‐6) are sensitive to these conditions [195].

Experimental studies indicate that unfavorable environmental conditions may reduce the efficacy of postbiotics. For example, under hypoxic conditions in shrimp, the immunomodulatory effect of *Pediococcus*‐based postbiotics has been observed to weaken [155, 196].

At the same time, numerous studies confirm their beneficial effects. In rainbow trout (*O. mykiss*), reduced mortality during *Yersinia ruckeri* infection and increased resistance to enteric redmouth disease have been reported [197]. In tilapia (*O. niloticus*), the application of postbiotics was accompanied by increased leukocyte, lysozyme, and immunoglobulin activity, as well as reduced IL‐6 and TNF‐α levels [198].

In Atlantic salmon (*S. salar*), IgM levels increased by up to 37% [161, 199], while yeast‐derived postbiotics enhanced resistance to *A. hydrophila* [200, 201]. In white shrimp (*Litopenaeus vannamei*), the application of *Pediococcus pentosaceus* PP4012 resulted in increased body weight and survival following *V. parahaemolyticus* infection, accompanied by enhanced immune protection [202, 203]. In common carp (*Cyprinus carpio*), increased complement system activity and phagocytosis were observed, contributing to enhanced resistance to infections. In addition, lysozyme activity was considered a stable indicator of nonspecific immune defense.

In recent years, the use of functional feeds with immunomodulatory properties has become a key research direction in aquaculture, aimed at improving fish resistance and maintaining stable physiological status (for review, see Muneeb et al. [204], and in particular Chapter 5 and Table 1, and Marmelo et al. [205] and Abdul Kari et al. [206]). In this context, the intestine is regarded as the main target organ, since mucosa‐associated lymphoid tissue (MALT), including the gut‐associated lymphoid tissue (GALT) system, plays a key role in coordinating local and systemic immune responses. It ensures antigen presentation, T‐cell differentiation, the production of effector molecules and cytokines, and interactions between the intestinal microbiota and the host immune system [207, 208].

Among the bioactive components of functional feeds, the strain *Lpb. plantarum* L‐137 may be of particular importance. It interacts with pattern recognition receptors (PRRs) through lipoteichoic acid, thereby activating immune signaling pathways [209]. Clinical studies confirm its ability to improve immune function, reduce the incidence of infections, and exert beneficial effects on metabolic processes (see, for instance, Hirose et al. [210]). The heat‐killed form HK L‐137 induces the expression of key immunological biomarkers, including IFN‐γ and IL‐12 [211].

Studies on Asian seabass (*L. calcarifer*) have shown that *B. subtilis*‐based supplements (CPP) increase immunoglobulin levels and phagocytic activity [188]. Enhanced expression of TNF‐α, IL‐6, and IL‐1β, as well as pattern recognition receptors (NLRC3, TLR22, MDA5), was also observed, accompanied by increased resistance to *Streptococcus iniae* [188].

Thus, the body of experimental evidence confirms that postbiotics exert a comprehensive beneficial effect on the immunological biomarkers of aquatic organisms. This is manifested in the activation of innate and humoral immunity, reduction of inflammatory responses, and increased resistance to infectious diseases, providing a scientific rationale for their broad application in modern aquaculture.

#### 4.6.3 Metabolic Dysfunction and Biomarkers of Health in Aquaculture

Metabolic dysfunction is a complex pathophysiological condition characterized by disruption of the balance of energy, carbohydrate, lipid, and protein metabolism in the organism. Under aquaculture conditions, this state develops predominantly under the influence of intensive farming systems, high stocking density, unfavorable water‐quality parameters, hypoxia, and unbalanced diets. These factors lead to lipid accumulation in the liver, development of steatosis, increased oxidative stress, and reduced immune function [212].

A key pathogenetic mechanism of metabolic disorders is the imbalance between energy intake and energy expenditure. Diets with high carbohydrate and lipid content activate glycolysis and lipogenesis, promoting the conversion of excess glucose into triglycerides. This results in fat accumulation in hepatocytes and metabolic stress [213].

Metabolic biomarkers are widely used for the objective assessment of metabolic status. These molecular indicators reflect carbohydrate, lipid, and energy metabolism, liver function, and oxidative stress. In aquaculture, they are conventionally classified into the following functional groups: glycemic biomarkers, lipid profile, hepatic enzymes, indicators of energy metabolism, and markers of oxidative stress.

Among glycemic biomarkers, blood glucose level is of key importance. Hyperglycemia is associated with impaired insulin signaling, reduced energy utilization, and activation of lipogenesis. This condition is particularly characteristic of intensively farmed species, such as tilapia and carp, and is regarded as an early marker of hepatic steatosis.

Lipid biomarkers are among the most important indicators of metabolic health and include triglycerides (TG), total cholesterol (TC), high‐density lipoproteins (HDL), and low‐density lipoproteins (LDL). An increase in TG and LDL, accompanied by a simultaneous decrease in HDL, is considered a characteristic sign of metabolic dysfunction and impaired lipid metabolism (for a generic review, see, for instance, Yang et al. [214]).

Hepatic enzymes ALT, AST, and ALP are used to assess liver function. Their elevation in blood plasma indicates hepatocellular injury, lipid infiltration, and toxic stress [215].

Biomarkers of oxidative stress represent an important component of metabolic dysfunction. Intensive aquaculture is accompanied by an imbalance between the production of reactive oxygen species (ROS) and the activity of the organism's antioxidant system, leading to oxidative stress [59, 216]. Increased ROS levels cause membrane damage, lipid peroxidation, protein denaturation, and DNA damage, which are accompanied by disruption of metabolic processes, reduced immune resistance, growth suppression, and increased susceptibility to infections [217]. The liver, intestine, and gills are the most vulnerable organs.

In response, the antioxidant system is activated, with superoxide dismutase (SOD), glutathione peroxidase (GPx), and catalase (CAT) serving as its key enzymes. These enzymes ensure the sequential neutralization of ROS: SOD converts the superoxide radical into hydrogen peroxide, which is then decomposed by GPx and CAT into water and oxygen.

A decrease in SOD, GPx, and CAT activity indicates weakened antioxidant defense, whereas an increase reflects an adaptive response in the organism and enhanced resistance to stress. Therefore, these enzymes are widely used as functional biomarkers in aquaculture. Another important marker is malondialdehyde (MDA), a classic indicator of lipid peroxidation. An increase in its level reflects the extent of membrane damage and the intensity of oxidative stress.

Recent studies show that functional feeds, including probiotics, postbiotics, and paraprobiotics, effectively modulate the antioxidant system by increasing the activity of SOD, GPx, and CAT while reducing MDA levels. This effect is associated with activation of the Nrf2/ARE signaling pathway, reduced inflammation, and stabilization of mitochondrial function.

Postbiotics based on lactobacilli and bacilli increase SOD and CAT activity in liver and intestinal tissues [11, 218], as well as enhance GPx activity, which protects cell membranes from oxidative damage. It has been established that *Bifidobacterium*‐based postbiotics reduce MDA levels by 15–25% [201, 219]. In white shrimp (*Litopenaeus setiferus*) receiving *P. pentosaceus*‐based postbiotics, a significant reduction in hepatic MDA was observed [202], confirming the protection of cell membranes and increased resistance to oxidative stress. Postbiotics derived from *Levilactobacillus brevis* BK3 exhibit pronounced antioxidant activity, neutralizing DPPH radicals by 30.97% and ABTS radicals by 46.65% [118]. They contribute to the stabilization of the intestinal microbiota, the reduction of free radical levels, and the maintenance of metabolic and immune homeostasis. Supplements based on *S. putrefaciens* and *Bacillus* spp. demonstrated improved metabolic stability in tilapia and salmon [201].

Thus, postbiotics are a promising class of biologically active, microbially derived compounds that can influence key physiological processes in aquatic organisms. The combined use of inflammatory, immunological, and metabolic indicators provides a comprehensive and objective basis for evaluating their biological efficacy. The data indicate that postbiotics reduce systemic inflammation, enhance innate and adaptive immune responses, and restore metabolic homeostasis, including by lowering oxidative stress through modulation of antioxidant defense mechanisms. The identified biomarkers—CRP, IL‐6, TNF‐α, immunoglobulins, enzymatic activity, and oxidative stress indicators (MDA, SOD, CAT, GPx)—not only reflect improvements in physiological status but also serve as quantifiable, relevant parameters for monitoring the efficacy of postbiotic preparations. Taken together, these findings highlight the key role of biomarker‐based approaches in expanding the application of postbiotics and substantiate their use as a sustainable and effective strategy for improving health, disease resistance, and productivity in aquaculture systems.

### 4.7 Marker Metabolites in Postbiotic Preparations for Aquaculture

From the perspective of modern microbiology and feed analytics, there is no single universal “primary” biomarker for postbiotics in aquaculture. This is expected: according to the ISAPP consensus definition [220], postbiotics are preparations of inactivated microorganisms and/or their components that must confer a demonstrated benefit to the host; consequently, different types of postbiotics will carry different “chemical mediators of effect.” In aquaculture, the beneficial effects of postbiotic supplements have already been demonstrated in hybrid sturgeon, carp, rainbow trout, and marine fish, although in most studies, the biological effects are described in greater detail than the chemical composition of the product batch. This leads to a key practical conclusion: quality control requires not a single marker but a modular panel that simultaneously confirms identity, the putative mechanism of action, the absence of viable cells, and molecular safety [221].

Based on the available evidence, the strongest candidates as biomarkers of efficacy/quality include short‐chain fatty acids and other organic acids, bacteriocins and bacteriocin‐like peptides, cyclic lipopeptides of *Bacillus*, amicoumacin A, lipoteichoic acids, peptidoglycan markers such as muramic acid, CpG‐rich bacterial DNA, and exopolysaccharides. For yeast‐fermentation‐derived postbiotics, additional multimetabolomic markers are relevant, including nucleotides/nucleosides, free amino acids, short peptides, and organic acids. The specificity of these classes differs: for claimed antimicrobial functionality, strain‐associated peptides and *Bacillus* metabolites are the most specific; for immunomodulation and “inactivated biomass,” lipoteichoic acids, peptidoglycan, and bacterial DNA are particularly relevant; whereas for general fermentation activity, organic acids and the metabolomic fingerprint are the most informative [107].

From an analytical perspective, an optimal scheme would include targeted LC–MS/MS or UPLC–MS/MS for bacteriocins, amicoumacin A, lipopeptides, nucleotides, and part of the organic acids; HILIC–MS after acid hydrolysis for quantitative assessment of peptidoglycan markers; 1‐butanol extraction combined with hydrophobic interaction chromatography and NMR/LC–MS for lipoteichoic acids; HPAEC‐PAD together with SEC‐MALS and NMR for exopolysaccharides; PMA‐qPCR/ddPCR to confirm the absence of viable cells, alongside dedicated qPCR panels for ARG and virulence genes in preserved DNA. For complex products, especially yeast fermentation‐derived cultures and multicomponent supernatants, targeted methods should be complemented by untargeted fingerprinting using HILIC/RP‐HRMS and/or ^1^H‐NMR, guided by validation and minimum reporting recommendations from ICH [222], AOAC INTERNATIONAL [114], and metabolomics reporting consensus statements [223].

Threshold values for finished aquaculture postbiotic products are rarely specified in the literature. Exceptions mainly concern functional assays of individual molecules: for example, for nisin Z against Lactococcus garvieae, MIC and MBC values of 5 AU/mL and 10 AU/mL, respectively, have been reported, while the activity of amicoumacin A against different Vibrio species varies by orders of magnitude; for PMA‐qPCR in probiotic matrices, limits of quantification (LOQ) and working ranges are also well characterized. However, these do not yet constitute universal release specifications for a postbiotic product. Therefore, at the current stage, the most scientifically sound and practically feasible approach is to combine absolute targeted measurements, where available, with relative “reference‐lot” acceptance criteria for the remaining markers [107].

#### 4.7.1 Rationale for Biomarker Sele

This review employs a broad yet strictly practical definition of an aquaculture postbiotic: inactivated microbial biomass, cellular components, cell‐free supernatants, or fermentation‐derived post‐products that have demonstrated benefits for fish or crustaceans, including immunomodulation, growth promotion, pathogen resistance, gut health, stress resilience, or indirect environmental safety. This approach aligns with the ISAPP consensus and reflects the terminology commonly used in aquaculture literature, where terms such as postbiotic, paraprobiotic, heat‐killed, and fermentation‐derived product are often used interchangeably [221].

The evidence base in aquaculture is now sufficient to allow a substantive discussion of biomarkers. In hybrid sturgeon, the feed additive HWF, described as a paraprobiotic/postbiotic, improved growth performance and intestinal microbiota function; in carp, SWF concentration reduced inflammation and oxidative stress under a high‐fat diet; in rainbow trout, a *Weissella cibaria*‐based postbiotic increased survival after *Yersinia ruckeri* challenge by approximately 20.66%; and in juvenile gilthead seabream, postbiotics derived from *Vibrio proteolyticus* DCF12.2 improved intestinal integrity, microbiota composition, and immune responses. At the same time, these studies reveal a major gap: feed dose is typically standardized, whereas the molecular profile of the active principle is not. This alone provides strong justification for considering chemical‐analytical standardization a central unresolved challenge [224].

From a regulatory perspective, the rationale is equally clear. Under EFSA approaches [125] and European feed additive regulations, product characterization, evidence of efficacy, and the absence of adverse effects for animals, humans, and the environment are required. For microbial ingredients, particular emphasis is placed on microbial identification and characterization, as well as safety assessment. Although most of these documents were developed primarily for live microbial feed additives, the same framework is arguably even more relevant for postbiotics: the absence of viability does not exempt them from the need to control chemical identity, residual DNA, antimicrobial resistance markers, and batch‐to‐batch consistency [143].

These considerations lead to a simple rule for selecting biomarker candidates: a good postbiotic biomarker should satisfy at least two of four criteria—it should be linked to a mechanism of action, be analytically measurable in formulation, be sufficiently specific to the given product type, and be regulatory relevant as a marker of safety or identity. The best biomarker panels meet all four criteria simultaneously [133].

#### 4.7.2 Candidate Biomarkers in Postbiotic Preparations

The most substantiated candidates are summarized below (Table 2). We distinguish between universal quality/safety markers and strain‐ or platform‐specific efficacy markers. This distinction is more meaningful than attempting to identify a single “magic molecule” applicable to all postbiotics [101].

In practice, Table 2 leads to another important conclusion. Organic acids are convenient and inexpensive, but by themselves, too nonspecific. Bacteriocins, amicoumacin A, and *Bacillus* lipopeptides, by contrast, are highly informative for antimicrobial preparations but unsuitable as universal markers across all postbiotics. Lipoteichoic acids (LTA), peptidoglycan, and bacterial DNA form a logical core for inactivated cellular preparations because they characterize the material itself and help explain immunological activity. Exopolysaccharides (EPS) and the yeast metabolome are particularly important for products making gut health and barrier support claims [225].

It should also be emphasized that in some aquaculture postbiotic preparations, efficacy is likely multicomponent rather than attributable to a single molecule. In the *W. cibaria* trout model, antibacterial activity decreased after protease treatment and heat exposure but did not disappear completely; the authors themselves discussed possible contributions from protein‐like molecules, organic acids, water‐soluble polymers, and hydrogen peroxide. This implies that for such products, single‐analyte QC is insufficient: at a minimum, one peptide/protein‐class marker, one marker of the acidic fraction, and one global fingerprint are required [197].

#### 4.7.3 Analytical Determination and Validation

For postbiotics in aquaculture, a two‐tier analytical framework appears most appropriate. The first tier is targeted: predefined molecular markers are quantitatively measured by product type and the claimed mechanism of action. The second tier is untargeted fingerprinting: HRMS and/or NMR capture the multidimensional batch profile, enabling monitoring of batch drift, extrusion‐related degradation, storage‐related changes, and deviations from a reference lot. This combination aligns most closely with formal analytical validation principles and current recommendations for metabolomics reporting [194].

Several clarifications are essential. First, the validation metrics in the table have largely been generated using related matrices, milk, bacterial cultures, probiotic blends, isolated cell components, and food yeasts, rather than in finished aquafeeds. These metrics cannot be transferred automatically; each aquafeed premix requires matrix‐matched validation in accordance with Q2(R2) principles, including selectivity, linearity, accuracy, precision, recovery, stability, carryover, and robustness, and the LOD/LOQ values must be justified [223].

Second, for complex postbiotics, especially cell‐free supernatants and yeast‐fermentation‐derived products, a single targeted assay is usually insufficient. In such cases, an untargeted workflow is appropriate: separate polar and semi‐polar extracts; HILIC‐HRMS for acids, amino acids, and nucleotides; RP‐HRMS for peptides and lipopeptides; pooled QC every 5–10 injections; extraction blanks; internal standards; and feature annotation according to MSI/MRF confidence levels. ^1^H‐NMR is particularly useful as an orthogonal platform for major organic acids and for detecting large batch‐to‐batch differences [226].

Third, because aquafeeds often undergo thermal processing, in‐process stability testing must be incorporated. For a *W. cibaria* postbiotic product, heating has been shown to reduce antibacterial activity without eliminating it entirely, making post‐extrusion control essential. Otherwise, releasing data for the starting preparation does not guarantee activity in the final feed [222, 227].

### 4.8 Comparative Evaluation and Priority Panel

When candidate biomarkers are compared across specificity, sensitivity, practicality, cost, and regulatory relevance, the picture becomes fairly clear. For antimicrobial postbiotics, strain‐specific potency markers rank highest: nisin/garvicins/weissellicin for LAB‐derived products, and amicoumacin A or lipopeptide‐sum (LP‐sum) for *Bacillus*‐derived products. These correlate best with the direct claim of “pathogen inhibition.” Their weakness is poor transferability across platforms and strains: what is ideal for an anti‐Vibrio Bacillus preparation may be irrelevant for heat‐killed *Lactiplantibacillus* spp. or yeast SCFP products [107].

For immunomodulatory inactivated bacterial preparations, LTA, peptidoglycan surrogates, and CpG‐rich DNA appear to be the strongest candidates. Here, platform specificity is lower than with bacteriocins, but mechanistic plausibility is much stronger, especially when an immune effect is claimed without a direct bactericidal claim. LTA in L‐137 has been linked to IL‐12 induction; CpG motifs are immunologically active in fish and shrimp; and muramic acid provides a practical quantitative surrogate for total immunoactive cellular material [225].

For gut health and barrier support claims, EPS and organic acids are the most logical candidates, whereas for yeast postbiotics, multicomponent metabolomic panels that include nucleotides, amino acids, and organic acids are preferable. However, this is precisely where single‐molecule specificity is weakest. Therefore, for such products, I consider it more appropriate to use either an EPS structural panel or an untargeted fingerprint combined with 2–4 sentinel markers, rather than a single analyte [125].

From a safety and regulatory‐suitability perspective, two indicators stand out above all others: the absence of viable cells and the absence of undesirable genetic markers. These parameters are the easiest to interpret for manufacturing release decisions and align most closely with the regulatory philosophy for microbial feed products (Table 5). This is particularly important in aquaculture, where the environmental dimension of antimicrobial resistance (AMR) cannot be ignored [228].

**TABLE 5:** Recommended priority biomarker panel for quality control and efficacy studies (examples).

Priority	Marker/Panel	Applicable products	What it addresses	Proposed threshold or decision rule	Specificity	Practicality/Cost	Regulatory relevance	Sources/ Comments
Safety core	Absence of viable cells	All postbiotics	Confirms inactivation; reduces risk of uncontrolled release of live microorganisms	PMA‐qPCR LOQ around 10^3^ CFU/mL; for product release—viable signal below validated LOQ + absence of growth in enrichment culture test	High	High/Low–Medium	Very high	[229]
Safety core	ARG/virulence gene screen in residual DNA	All postbiotics, especially bacterial	Control of molecular safety and environmental risk	Target ARG/virulence markers not detected by selected panel; for complex products—confirmatory WGS/amplicon analysis where appropriate	High	Medium/Medium	Very high	[230]
Potency core for LAB antimicrobial claims	Specific bacteriocin or bacteriocin‐family marker	LAB postbiotics with pathogen inhibition claims	Most direct chemical equivalent of antimicrobial activity	Literature: for nisin Z against *L. garvieae*, MIC 5 AU/mL and MBC 10 AU/mL; no formulation threshold available, therefore internal release specification + parallel bioassay recommended	High	Medium/Medium–High	Medium–High	Functional thresholds exist for purified activity, but not for finished products [108, 231]
Potency core for *Bacillus* antimicrobial claims	LP‐sum and/or amicoumacin A	*Bacillus*‐derived postbiotics	Potential principal mediator of the anti‐*Vibrio* effect	Amicoumacin A active range against *Vibrio* ∼0.5–64 µg/mL depending on strain;	High	Medium/Medium–High	Medium	[114]
Immune claim core	Muramic acid + LTA signature	Heat‐killed Gram+ biomass, paraprobiotic/postbiotic blends	Quantifies immunoactive cellular cargo and supports mechanistic plausibility	No threshold established;	Medium–High	Medium/Medium–High	High	[225, 232]
Conditional gut‐health core	EPS concentration + MW distribution + monosaccharide pattern	EPS‐rich LAB/*Bacillus* products	Strongest link to mucosal/barrier claims	No threshold established;	Medium	Medium/Medium–High	Medium	[125]
Conditional core for yeast postbiotics	Nucleotides + free amino acids/short peptides + organic acid panel	SCFP and related yeast fermentation‐derived products	Describes nutritionally active metabolome, often not captured in proximate analysis	No threshold established;	Medium	Medium/Medium	Medium	[128]

In practical algorithmic terms, this implies the following. For release QC, every postbiotic should include at a minimum four mandatory modules: (A) absence of viable cells; (B) a molecular safety screen; (C) one or two identity markers, depending on the product platform; (D) one potency marker, depending on the claim.

For efficacy studies, these should be supplemented by verification that marker concentration actually predicts biological effect in the target species. Without such a correlation, even elegant chemistry remains merely a batch characteristic rather than a true biomarker of benefit [227].

For claims such as pathogen inhibition, the efficacy study design should link chemical markers to a challenge model against a relevant pathogen and at least one early host endpoint. For gut health claims, markers should be linked to intestinal integrity, shifts in the microbiota, and mucosal histology. For immune modulation claims, they should be linked to early transcriptional and/or serum indicators of innate immunity. This bridge between formulation biomarkers and host biomarkers remains largely underdeveloped, which is precisely one reason why field translation has progressed slowly.

### 4.9 Standardization Gaps

The central scientific problem in the current literature is not the absence of positive results, but the disconnect between biological phenotypes and chemical‐analytical standardization. In HWF, SWF concentration, W. cibaria postbiotic, and several yeast‐derived products, biological effects have been described quite convincingly, yet product composition is either only partially disclosed or effectively replaced by a description of manufacturing origin. Commercial yeast products are especially illustrative in this regard: guaranteed proximate analysis is disclosed, but not the panel of active metabolites that could serve as a true QC tool [224].

This leads directly to several concrete standardization needs. First, every product portfolio should include a reference lot concept: a qualified reference batch characterized not only by manufacturing process but also by untargeted fingerprinting, a targeted analytical panel, and functional bioassays. Without this, any statement such as “0.5% postbiotic in feed” or “1 g/kg SCFP” remains a black box, and interlaboratory reproducibility becomes largely accidental [227].

Second, standardization should be **modular rather than universal**. A robust international standard should define not one mandatory analyte, but a mandatory panel structure consisting of: (A) a safety module; (B) an identity module; (C) a potency module; (D) a storage/process stability module.

The specific analyte(s) within each module should depend on the platform type: LAB‐CFS, *Bacillus* metabolite products, heat‐killed Gram‐positive preparations, or yeast‐fermentation‐derived products. This is far more realistic than searching for a single marker applicable in all cases [101].

Third, matrix‐matched validation specific to aquafeeds is needed. At present, the best precision and LOQ metrics have been reported for milk, bacterial supernatants, purified cell components, and food yeasts. For the aquafeed industry, this is insufficient: lipids, mineral additives, binding agents, and extrusion‐associated thermal processing fundamentally alter extraction behavior and recovery. Accordingly, transfer validation for premix, mash feed, extruded pellets, and coated pellets should become standard practice rather than an optional refinement [223].

Fourth, interlaboratory ring validation is needed. Metabolomic and HRMS profiles without interlaboratory QA/QC are poorly suited for regulatory decision‐making. Here, minimal reporting principles and QA/QC frameworks already developed by the metabolomics community could be highly valuable, although they have been scarcely implemented in the feed postbiotic sector [226].

Finally, a **One** **Health perspective** is particularly important for aquaculture. Replacing live probiotics with postbiotics may indeed reduce the risk of viable microbial release, but residual DNA containing ARG markers, where present, should not be overlooked. For this reason, we consider ARG screening a safety marker that should be included in the mandatory core of standardization earlier than many fashionable metabolomic indicators.

Thus, the best current strategy is not to search for a single “aquaculture postbiotic biomarker,” but to construct type‐specific panels. For cellular Gram‐positive postbiotics, the core would consist of LTA + PG + DNA/safety markers; for LAB supernatants, organic acids + a bacteriocin marker, and, where appropriate, EPS; for Bacillus‐derived products, lipopeptide/amicoumacin potency markers; and for yeast SCFP products, a multimetabolite fingerprint comprising nucleotides, amino acids, and organic acids. Because absolute threshold values are still largely absent from the literature, the most appropriate approach until international standards emerge is a reference‐lot strategy, supported by rigorous analytical validation and an obligatory linkage between the chemical marker panel and host efficacy data [101].

## 5 Conclusion: Probiotics, Postbioitics, and Complementary approaches

The concept of probiotics emerged over a century ago. Pioneers of applied microbiology proposed and described the health‐promoting benefits of live microorganisms.

Probiotics have been and will continue to make a significant contribution to improving health in aquaculture. Probiotics have undeniable potential to improve aquaculture sustainability, but their benefits are not universal and not guaranteed. Variability in strain performance, host response, environmental conditions, and product quality highlights the need for a more critical and evidence‐based health management strategy in aquaculture. Probiotics should not be viewed as a final choice, but rather as one component of a broader, multifaceted aquatic animal health management strategy.

The evolution of our understanding of life systems, the role of microorganisms, and the importance of specific metabolites in health promotion mechanisms has been intensively studied in recent decades and has led to the concept of health promotion formulated within the One Health initiative. A deeper understanding of the mechanisms, taking into account ecological balance, the specifics of microbial metabolism and physiology, and the need for more complex and multi‐targeted approaches to improving health and economic performance in aquaculture, should inform future research. Priority should be given to understanding the mechanisms, long‐term field testing, and a rigorous regulatory framework to ensure the scientific validity and practical effectiveness of probiotic use.

However, thanks to advances in knowledge, the application of new analytical methods, and the accumulation of compelling scientific evidence, we can now capitalize on the benefits of probiotics, assess the limitations of their use and potential side effects, and seek a more holistic approach to health management within the framework of the “One Health” concept, including in aquaculture.

Postbiotics are perhaps one of the most discussed and most promising alternatives to traditional probiotics in aquaculture. Simply put, postbiotics do not require colonization or survival in the gastrointestinal tract, eliminating the risks associated with horizontal gene transfer, environmental disruption, or opportunistic pathogenicity. Their composition can (and should) be standardized, ensuring strictly controlled dosage and predictable biological effects in various production systems. Postbiotics contain bioactive compounds such as short‐chain fatty acids, peptides, exopolysaccharides, bacteriocins, and other bioactive peptides, as well as cell wall components that modulate the immune response, improve barrier function, and increase antioxidant capacity in fish and other aquatic organisms that are part of aquaculture. Due to their stability under changing environmental conditions, postbiotics integrate well into functional feeds and manufacturing protocols, where live cultures often prove ineffective. Postbiotic studies conducted by independent groups of investigators demonstrate improved growth, stress resistance, and disease resistance in eukaryotic organisms, generally comparable to or superior to probiotics.

It seems to us that probiotics originating from aquatic organisms are often more effective in aquatic hosts than strains derived from terrestrial environments (see, for instance, Wuertz et al. [233], Gao et al. [234], Katariya et al. [235]). If this observation is correct, an interesting question emerges: if postbiotics are to complement or even replace probiotics in aquaculture, what actually constitutes the “aquatic‐specific” component responsible for these host‐adapted effects?

We hypothesize that the beneficial effects observed with aquatic host‐derived probiotics may not be attributable to a single metabolite, but rather to a characteristic “postbiotic fingerprint” consisting of multiple bioactive components. Such a fingerprint could include metabolites, extracellular vesicles, exopolysaccharides, cell‐envelope components, signaling molecules, and other host‐adapted microbial products.

From this perspective, an important future research direction may be to identify which postbiotic components are responsible for the host‐specific effects observed in aquatic organisms and whether these differ from the products generated by terrestrial or human‐derived probiotic strains.

Compared to over a century of research and nearly a century of industrial production and use of probiotics, the field of postbiotic research and use in human and animal health is relatively young. Postbiotic research began in the 1990s, possibly with the work of Matsuzaki et al., who first reported the beneficial immunomodulatory effect of heat‐killed probiotics *Lacticaseibacillus* (formerly *Lactobacillus*) *casei* Shirota in animal studies [236].

Certainly, the use of postbiotics in aquaculture has increased significantly over the past 5 years, largely due to the recognition that their use avoids some of the practical problems associated with probiotics. Recent studies have demonstrated that postbiotics can: (A) Improve gut barrier integrity and microbiome diversity in fish; (B) Reduce pro‐inflammatory signalling after immune challenge; (C) enhance disease resistance and survival in species like rainbow trout and shrimp [86]. Postbiotics are documented as delivering (A) Antibacterial and anti‐biofilm activity; (B) Immune activation (NO, cytokines, leukocyte proliferation), and (C) Improved resistance to pathogens in experimental fish models [186].

Overall, it seems that postbiotics may offer similar functional benefits to probiotics but with greater stability and safety, making them attractive for feed formulation in aquaculture systems [237]. Most current work focuses on cell‐free supernatants or heat‐killed probiotics (especially *Bacillus* spp. and lactic acid bacteria). An emerging concept is that microbial metabolites may influence epigenetic regulation in fish (DNA methylation, histone acetylation) via: (A) SCFAs influencing histone acetylation, (B) QS molecules affecting immune gene expression, and (C) Microbial metabolites influencing stress response pathways. Recent work links postbiotics and epigenetic regulation of stress, growth, and immunity in fish [237].

Fish health strongly depends on mucus layers and epithelial integrity. Postbiotics may: (A) Stimulate goblet cell proliferation; (B) Strengthen tight junctions, and (C) Regulate mucin production. Some experiments already show improved epithelial gene expression after postbiotic feeding [86].

Notably, postbiotics may find their niche in anti‐virulence rather than antimicrobial applications. Instead of killing pathogens, postbiotics may act to: (A) Disrupt QS; (B) Inhibit biofilm formation, and (C) Suppress virulence gene expression. This strategy is particularly attractive for *Vibrio* control in shrimp systems [238].

While most studies focus on the use of postbiotics for feed supplementation, there is also recognised potential for: (A) Water column conditioning; (B) Biofloc modulation; and (C) Suppression of pathogenic vibrios in ponds. This aligns with microbiome management of aquaculture systems, not just the host [239].

Regarding the industrial production of postbiotics for human consumption, relevant regulations must be followed, such as avoiding the use of animal‐based nutrient sources for the propagation of microorganisms. Similarly, appropriate regulations must be followed when producing postbiotics for animals in general and for use in aquaculture in particular. However, given the growing demand for sustainable development and efficient waste management in agriculture, the possibility of using food and agricultural waste as nutrient‐rich substrates for microbial growth should be considered [240]. In the context of aquaculture waste valorization, it may be of interest to focus on the fermentation of fish processing waste, algae residues, and other agricultural by‐products to produce postbiotic metabolites for feed additives.

Another concept that is gaining increasing importance for application in humans and other animals is what we would call “precision postbiotics.” As already mentioned, according to the ISAPP definition, postbiotic preparations contain non‐living microorganisms and/or unpurified products of their fermentation. However, intelligent selection of source microorganisms, fermentation conditions, and their inactivation procedures can facilitate the production of postbiotics with specifically enhanced properties (this has been recently discussed here—see Chikindas et al. [191]. This will enable the production of precision postbiotics enriched with essential substances such as bacteriocins, short‐chain fatty acids, extracellular vesicles, peptidoglycan fragments, quorum‐sensing inhibitors, etc., which can be delivered to their site of action as targeted functional ingredients.

And while we are on the topic of precision postbiotics, it is worth noting that their use to address specific larval development needs is becoming an exciting and highly important challenge, especially bearing in mind that larval mortality is clearly a huge problem. The potential precision applications may include immune priming in larvae, gut colonization modulation, and morphogenesis signalling. This is a clearly underexplored area since larval microbiome work is still emerging (see, for instance, Paralika and Makridis [241]).

**FIGURE 1:**
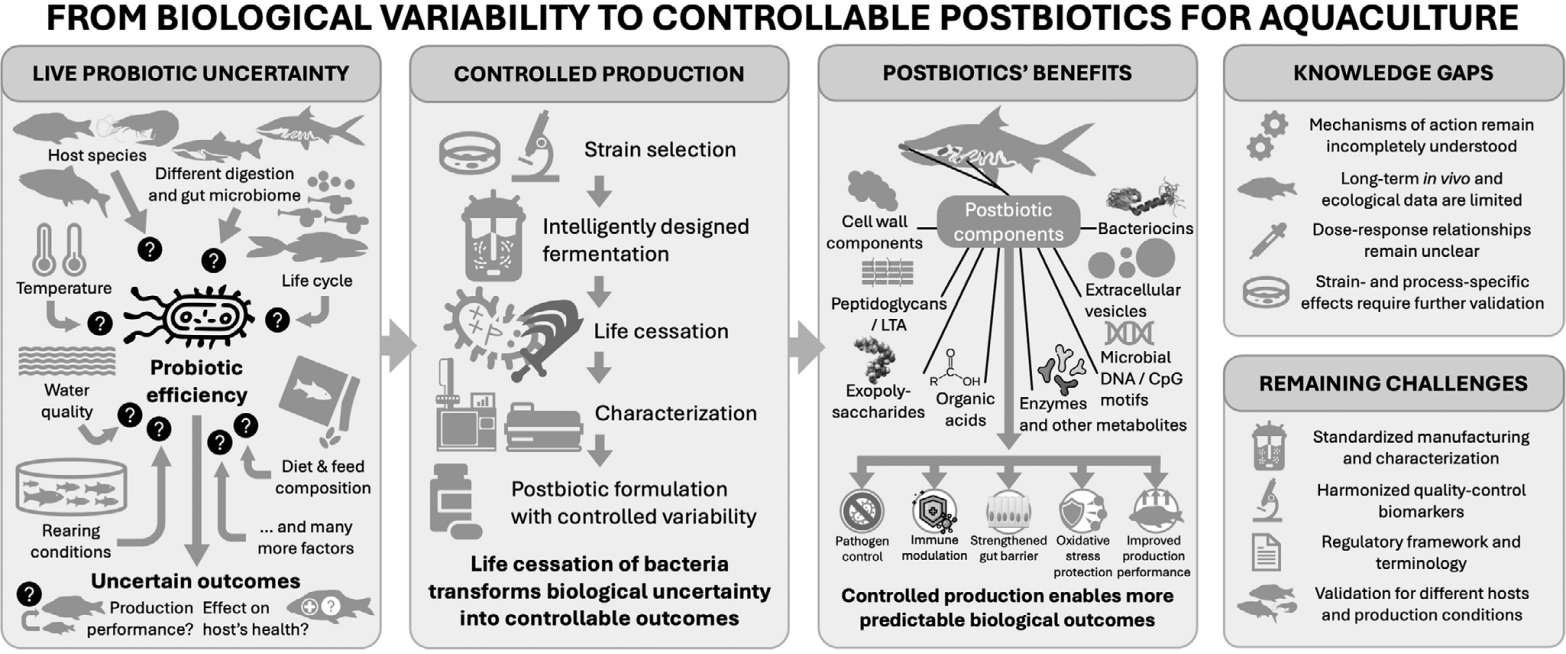
From biological variability to controllable postbiotics for aquaculture. The scheme illustrates the transition from the variable performance of live probiotics to the more standardized production and application of postbiotics. Probiotic efficacy is influenced by host species, digestive physiology and gut microbiota, life stage, temperature, water quality, rearing conditions, diet, and other interacting factors, resulting in uncertain effects on health and production performance. Postbiotic production involves progenitor‐strain selection, controlled fermentation, validated microbial inactivation, product characterization, and formulation with defined composition and variability. Because efficacy no longer depends on microbial survival or colonization, this approach can improve manufacturing reproducibility and product stability. Postbiotic preparations may contain inactivated cells and cell‐wall components, peptidoglycans and lipoteichoic acids, exopolysaccharides, extracellular vesicles, microbial DNA, enzymes, bacteriocins, organic acids, and other metabolites. These components may support pathogen control, immune modulation, intestinal barrier integrity, oxidative‐stress protection, and production performance. Remaining knowledge gaps include incomplete understanding of mechanisms, limited long‐term and dose‐response data, and strain‐ and process‐specific effects, while practical challenges include manufacturing standardization, harmonized quality control, regulatory clarity, and validation across hosts and production conditions.

From a strategic perspective, postbiotics should not be viewed as a “silver bullet” that will replace probiotics and other natural and nature‐derived health‐promoting products for humans and other animals. Moreover, it should be clearly understood that the mechanisms of action of probiotics often have a postbiotic basis, which makes these two health‐promoting tools closely interrelated. In fact, the future lies in complementary approaches, where postbiotics can and will be used alongside probiotics [117], bacteriophages [117], and other bioactive substances, or even synergize with antibiotics [243] to ensure the most effective and reproducible health effects on the target eukaryotic organism. Postbiotics represent a complementary strategy that may overcome several technological and safety limitations associated with live microbial administration, offering advantages in stability and safety, but still requiring strain‐specific validation, definition of active components, mechanistic evidence and dose/response studies (Figure 1).

To conclude, as regulatory frameworks increasingly focus on safety and reproducibility, postbiotics represent a scalable, low‐risk, and mechanistically transparent tool for the sustainable development of healthy aquaculture. Furthermore, we reiterate that postbiotics should not be viewed as a stand‐alone “magic bullet,” but rather as a key component of an integrated One Health system aimed at improving ecosystems of living organisms, such as those discussed in this review of aquaculture. The future of sustainable aquaculture lies in the use of precision postbiotics, tailored formulations created with selected bacterial strains, controlled fermentation and targeted inactivation protocols to address developmental stage‐specific challenges (e.g. larval immunization, mucus barrier protection and aquatic environment conditioning), as well as complementary strategies such as bacteriophages and functional feeds.

We propose that postbiotics represent not simply a safer substitute for probiotics, but a conceptual shift from administering living microorganisms toward engineering reproducible microbial functions. Aquaculture provides an ideal model in which to develop this transition because it exposes microorganisms to exceptionally diverse environmental conditions. Lessons learned in aquatic systems are therefore likely to inform precision postbiotic design in livestock, companion animals, and eventually human medicine.

## Author Contributions

Literature overview, Writing – original draft: MLC, DX, IVP, KOC, NAE, GAB, DAA, AP, SMM, OVM, LSS, JT, SDT; Figure design: IVP; Formating and editing: MLC, JT, SDT; Final revisions and validation: MLC, JT, SDT.

## Ethics Statement

The current manuscript is a critical overview of the existing literature and does not contain experiments involving animals or humans.

## Conflicts of Interest

L.S.S. has equity in Stellar Biotics, LLC. J.R.T has equity in BLIS Technologies. The other authors declare no conflicts of interest.

## References

[1] A. Amobonye , B. Pillay , F. Hlope , S. T. Asong , and S. Pillai , “Postbiotics: an Insightful Review of the Latest Category in Functional Biotics,” World Journal of Microbiology and Biotechnology 41, no. 8 (2025): 293, 10.1007/s11274-025-04483-8. PMC12317891 40751848

[2] C. E. Nash , The History of Aquaculture (Wiley‐Blackwell, 2011), 10.1002/9780470958971.

[3] K. Hua , J. M. Cobcroft , A. Cole , et al., “The Future of Aquatic Protein: Implications for Protein Sources in Aquaculture Diets,” One Earth 1, no. 3 (2019): 316–329, 10.1016/j.oneear.2019.10.018.

[4] F. A. O. Code of Conduct , “Code of Conduct for Responsible Fisheries,” Food and Agriculture Organization of the United Nations 1, no. 1 (2026): 1–55, https://www.fao.org/fishery/code/en.

[5] J. S. Mackenzie and M. Jeggo , “The One Health Approach—Why Is It So Important?,” Tropical Medicine and Infectious Disease 4, no. 2 (2019): 88, 10.3390/tropicalmed4020088. PMC6630404 31159338

[6] K. Laktuka , A. Kalnbalkite , L. Sniega , and K. Logins , “Towards the Sustainable Intensification of Aquaculture: Exploring Possible Ways Forward,” Sustainability 15, no. 24 (2023): 16952, 10.3390/su152416952.

[7] C. Hill , F. Guarner , G. Reid , et al., “The International Scientific Association for Probiotics and Prebiotics Consensus Statement on the Scope and Appropriate Use of the Term Probiotic,” Nature Reviews Gastroenterology & Hepatology 11, no. 8 (2014): 506–514, 10.1038/nrgastro.2014.66. 24912386

[8] S. H. Hoseinifar , M. Faheem , I. Liaqat , et al., “Promising Probiotic Candidates for Sustainable Aquaculture: an Updated Review,” Animals 14, no. 24 (2024): 3644, 10.3390/ani14243644. PMC11672650 39765548

[9] A. Gadhiya , S. Katariya , K. Khapandi , and D. Chhatrodiya , “Probiotics as a Sustainable Alternative to Antibiotics in Aquaculture: a Review of the Current State of Knowledge,” The Microbe 8, no. 1 (2025): 100426, 10.1016/j.microb.2025.100426.

[10] S. D. Todorov , J. M. S. Lima , J. E. V. Bucheli , I. V. Popov , S. K. Tiwari , and M. L. Chikindas , “Probiotics for Aquaculture: Hope, Truth, and Reality,” Probiotics and Antimicrobial Proteins 16, no. 6 (2024): 2007–2020, 10.1007/s12602-024-10290-8. 38801620

[11] S. Salminen , M. C. Collado , A. Endo , et al., “The International Scientific Association of Probiotics and Prebiotics (ISAPP) Consensus Statement on the Definition and Scope of Postbiotics,” Nature Reviews Gastroenterology & Hepatology 18, no. 9 (2021): 649–667, 10.1038/s41575-021-00440-6. PMC8387231 33948025

[12] Y. Luan , M. Li , W. Zhou , et al., “The Fish Microbiota: Research Progress and Potential Applications,” Engineering 29, no. 1 (2023): 137–146, 10.1016/j.eng.2023.01.011.

[13] L. Z. Auclert , M. S. Chhanda , and N. Derome , “Interwoven Processes in Fish Development: Microbial Community Succession and Immune Maturation,” PeerJ 12, no. 1 (2024): 17051, 10.7717/peerj.17051. PMC10981415 38560465

[14] A. D. Diwan , S. N. Harke , and A. N. Panche , “Host‐microbiome Interaction in Fish and Shellfish: an Overview,” Fish and Shellfish Immunology Reports 4, no. 1 (2023): 100091, 10.1016/j.fsirep.2023.100091. PMC10113762 37091066

[15] A. R. Wang , C. Ran , E. Ringø , and Z. G. Zhou , “Progress in Fish Gastrointestinal Microbiota Research,” Reviews in Aquaculture 10, no. 3 (2018): 626–640, 10.1111/raq.12191.

[16] P. S. Kim , N. R. Shin , J. B. Lee , et al., “Host Habitat Is the Major Determinant of the Gut Microbiome of Fish,” Microbiome 9, no. 1 (2021): 166, 10.1186/s40168-021-01112-4. PMC8325807 34332628

[17] S. Egerton , S. Culloty , J. Whooley , C. Stanton , and R. P. Ross , “The Gut Microbiota of Marine Fish,” Frontiers in Microbiology 9, no. 1 (2018): 873, 10.3389/fmicb.2018.00873. PMC5946678 29780377

[18] K. E. SULLAM , S. D. ESSINGER , C. A. LOZUPONE , et al., “Environmental and Ecological Factors That Shape the Gut Bacterial Communities of Fish: a Meta‐Analysis,” Molecular Ecology 21, no. 13 (2012): 3363–3378, 10.1111/j.1365-294X.2012.05552.x. PMC3882143 22486918

[19] H. Liu , X. Guo , R. Gooneratne , et al., “The Gut Microbiome and Degradation Enzyme Activity of Wild Freshwater Fishes Influenced by Their Trophic Levels,” Scientific Reports 6, no. 1 (2016): 24340, 10.1038/srep24340. PMC4829839 27072196

[20] X. Yang , Y. Xiao , X. Shi , et al., “Gut Microbiota and Metabolic Functions in Herbivorous Fish From Xisha Coral Reefs, China,” Marine Environmental Research 208 (2025): 107148, 10.1016/j.marenvres.2025.107148. 40220657

[21] T. Ofek , M. Lalzar , S. Laviad‐Shitrit , I. Izhaki , and M. Halpern , “Comparative Study of Intestinal Microbiota Composition of Six Edible Fish Species,” Frontiers in Microbiology 12, no. 1 (2021): 760266, 10.3389/fmicb.2021.760266. PMC8689067 34950115

[22] Z. Li , J. Lv , J. Chen , et al., “Comparative Study of Gut Content Microbiota in Freshwater Fish with Different Feeding Habits: a Case Study of an Urban Lake,” Journal of Fish Biology 106, no. 3 (2025): 823–835, 10.1111/jfb.15921. 39567260

[23] R. Xu , Y. Yu , and T. Chen , “Exploring the Dark Side of Probiotics to Pursue Light: Intrinsic and Extrinsic Risks to be Opportunistic Pathogens,” Current Research in Food Science 10, no. 1 (2025): 101044, 10.1016/j.crfs.2025.101044. PMC11999689 40235735

[24] B. Pan , X. Han , K. Yu , H. Sun , R. Mu , and C.‐A. Lian , “Geographical Distance, Host Evolutionary History and Diet Drive Gut Microbiome Diversity of Fish across the Yellow River,” Molecular Ecology 32, no. 5 (2023): 1183–1196, 10.1111/mec.16827. 36478318

[25] M. Soh , Y. C. Tay , C. S. Lee , et al., “The Intestinal Digesta Microbiota of Tropical Marine Fish Is Largely Uncultured and Distinct From Surrounding Water Microbiota,” npj Biofilms and Microbiomes 10, no. 1 (2024): 11, 10.1038/s41522-024-00483-w. PMC10876542 38374184

[26] R. Tang , J. Wang , X. Wang , et al., “Large‐scale Metagenomic Analysis Reveals Host Genetics Shapes Microbiomes in Wild Freshwater Fish Gut and Skin,” Cell Reports 45, no. 2 (2026): 116930, 10.1016/j.celrep.2026.116930. 41619209

[27] G. Lilli , C. Sirot , H. Campbell , D. Brophy , C. T. Graham , and I. F. George , “Geographic Origin and Host's Phylogeny Are Predictors of the Gut Mucosal Microbiota Diversity and Composition in Mediterranean Scorpionfishes (Scorpaena spp.),” Frontiers in Marine Science 10, no. 1 (2023): 112415, 10.3389/fmars.2023.112415.

[28] M. Sevellec , M. Laporte , A. Bernatchez , N. Derome , and L. Bernatchez , “Evidence for Host Effect on the Intestinal Microbiota of Whitefish (*Coregonus* sp.) Species Pairs and Their Hybrids,” Ecology and Evolution 9, no. 20 (2019): 11762–11774, 10.1002/ece3.5678. PMC6822036 31695886

[29] J. J. Minich , A. Härer , J. Vechinski , et al., “Host Biology, Ecology and the Environment Influence Microbial Biomass and Diversity in 101 Marine Fish Species,” Nature Communications 13, no. 1 (2022): 6978, 10.1038/s41467-022-34668-w. PMC9671965 36396943

[30] X. Jin , H. Zhu , Y. Shi , et al., “Host Hybridization Dominates over Cohabitation in Affecting Gut Microbiota of Intrageneric Hybrid *Takifugu* Pufferfish,” mSystems 8, no. 2 (2023): 0118122, 10.1128/msystems.01181-22. PMC10134855 36815841

[31] M. C. Piazzon , F. Naya‐Català , E. Perera , et al., “Genetic Selection for Growth Drives Differences in Intestinal Microbiota Composition and Parasite Disease Resistance in Gilthead Sea Bream,” Microbiome 8, no. 1 (2020): 168, 10.1186/s40168-020-00945-x. PMC7686744 33228779

[32] F. Naya‐Català , M. C. Piazzon , S. Torrecillas , et al., “Genetics and Nutrition Drive the Gut Microbiota Succession and Host‐Transcriptome Interactions through the Gilthead Sea Bream (*Sparus aurata*) Production Cycle,” Biology 11, no. 12 (2022): 1744, 10.3390/biology11121744. PMC9774573 36552254

[33] M. Ziab , S. R. Chaganti , and D. D. Heath , “The Effects of Host Quantitative Genetic Architecture on the Gut Microbiota Composition of Chinook Salmon (Oncorhynchus tshawytscha),” Heredity 131, no. 1 (2023): 43–55, 10.1038/s41437-023-00619-3. PMC10313681 37179383

[34] I. Z. Anka , T. Uren Webster , S. McLaughlin , et al., “Gut Microbiota Diversity Affects Fish Behaviour and Is Influenced by Host Genetics and Early Rearing Conditions,” Open Biology 15, no. 4 (2025): 240211, 10.1098/rsob.240211. PMC12001083 40237041

[35] S. B. Hansen , D. Bozzi , S. S. T. Mak , et al., “Intestinal Epigenotype of Atlantic Salmon (*Salmo salar*) Associates with Tenacibaculosis and Gut Microbiota Composition,” Genomics 115, no. 3 (2023): 110629, 10.1016/j.ygeno.2023.110629. 37100093

[36] G. Schwob , L. Cabrol , T. Saucède , K. Gérard , E. Poulin , and J. Orlando , “Unveiling the Co‐phylogeny Signal between Plunderfish *Harpagifer* spp. And Their Gut Microbiomes across the Southern Ocean,” Microbiology Spectrum 12, no. 4 (2024): 0383023, 10.1128/spectrum.03830-23. PMC10986581 38441978

[37] G. De Marco , T. Cappello , and M. Maisano , “Histomorphological Changes in Fish Gut in Response to Prebiotics and Probiotics Treatment to Improve Their Health Status: a Review,” Animals 13, no. 18 (2023): 2860, 10.3390/ani13182860. PMC10525268 37760260

[38] S. Fu , K. Qian , X. Tu , et al., “Comparative Analysis of Intestinal Structure, Enzyme Activity, Intestinal Microbiota and Gene Expression in Different Segments of Pufferfish (*Takifugu obscurus*),” Comparative Biochemistry and Physiology Part D: Genomics and Proteomics 52, no. 1 (2024): 101341, 10.1016/j.cbd.2024.101341. 39427531

[39] K. Zhu , M. Jiang , M. Yan , Y. Huang , T. Yang , and C. Zhu , “Characteristics and Functions of Different Intestinal Segments in Juvenile Greater Amberjack (Seriola dumerili),” Animals 15, no. 11 (2025): 1672, 10.3390/ani15111672. PMC12153913 40509138

[40] F. Jiao , L. Zhang , S. M. Limbu , et al., “A Comparison of Digestive Strategies for Fishes with Different Feeding Habits: Digestive Enzyme Activities, Intestinal Morphology, and Gut Microbiota,” Ecology and Evolution 13, no. 9 (2023): 10499, 10.1002/ece3.10499. PMC10495811 37706163

[41] Y. Liu , X. Li , Y. Li , J. Li , and S. Zhu , “Gut Microbiomes of Cyprinid Fish Exhibit Host‐species Symbiosis along Gut Trait and Diet,” Frontiers in Microbiology 13, no. 1 (2022): 941121, 10.3389/fmicb.2022.941121. PMC9396210 36016786

[42] Y. Liu , J. Cheng , Y. Xia , X. Li , Y. Liu , and P.‐F. Liu , “Response Mechanism of Gut Microbiome and Metabolism of European Seabass (*Dicentrarchus labrax*) to Temperature Stress,” Science of the Total Environment 813, no. 1 (2022): 151786, 10.1016/j.scitotenv.2021.152514. 34942265

[43] L. Chen , J. Ren , Y. Tian , et al., “Dynamics of Intestinal Organization and Microbiota during Early Development of *Procypris Mera* ,” Aquaculture 615, no. 1 (2026): 743599, 10.1016/j.aquaculture.2025.743599.

[44] Y. Zha , E. S. Lindström , A. Eiler , and R. Svanbäck , “Different Roles of Environmental Selection, Dispersal, and Drift in the Assembly of Intestinal Microbial Communities of Freshwater Fish with and without a Stomach,” Frontiers in Ecology and Evolution 8 (2020): 152, 10.3389/fevo.2020.00152.

[45] J. M. Amillano‐Cisneros , P. T. Hernández‐Rosas , B. Gomez‐Gil , et al., “Loss of Gut Microbial Diversity in the Cultured, Agastric Fish, Mexican Pike Silverside (*Chirostoma estor*: Atherinopsidae),” PeerJ 10, no. 1 (2022): 13052, 10.7717/peerj.13052. PMC8908885 35282279

[46] K. Roy , L. Kajgrova , L. Capkova , et al., “Synergistic Digestibility Effect by Planktonic Natural Food and Habitat Renders High Digestion Efficiency in Agastric Aquatic Consumers,” Science of the Total Environment 927, no. 1 (2024): 172105, 10.1016/j.scitotenv.2024.172105. 38556011

[47] E. M. Mkulo , L. Iddrisu , M. A. Yohana , et al., “Exploring Salinity Adaptation in Teleost Fish, Focusing on Omics Perspectives on Osmoregulation and Gut Microbiota,” Frontiers in Marine Science 12, no. 1 (2025): 1432104, 10.3389/fmars.2025.1432104.

[48] B. K. Singh , K. Thakur , H. Kumari , et al., “A Review on Comparative Analysis of Marine and Freshwater Fish Gut Microbiomes: Insights into Environmental Impact on Gut Microbiota,” FEMS Microbiology Ecology 101, no. 1 (2025): fiae169, 10.1093/femsec/fiae169. PMC11730441 39719366

[49] J. Li , S. Boonanuntanasarn , J. Kiatmontri , et al., “Effects of Dietary Salt and Water Salinity on the Dynamics of Major Gut Microbiota (*Cetobacterium*), Antioxidant Status, and Immune Response in Nile Tilapia,” Aquaculture 612, no. 1 (2026): 743235, 10.1016/j.aquaculture.2025.742114.

[50] C. Peng , H. Xue , J. Zhang , et al., “Salinity Modulates Gut Microbiota and Host Transcriptome Dynamics in Juvenile Euryhaline Fish Yellowfin Seabream (Acanthopagrus latus),” Aquaculture Reports 44, no. 1 (2025): 103075, 10.1016/j.aqrep.2025.103075.

[51] Y. Xiao , Z. Zhao , Y. Pan , et al., “Seawater Adaptation of Freshwater Cultured Rainbow Trout (*Oncorhynchus mykiss*): Insights into the Gut Microbiota and Tissue Gene Expression Profiles,” Aquaculture 612, no. 1 (2026): 743170, 10.1016/j.aquaculture.2025.742115.

[52] S. M. Morshed , Y.‐Y. Chen , C.‐H. Lin , Y.‐P. Chen , and T.‐H. Lee , “Freshwater Transfer Affected Intestinal Microbiota with Correlation to Cytokine Gene Expression in Asian Sea Bass,” Frontiers in Microbiology 14, no. 1 (2023): 1124151, 10.3389/fmicb.2023.1124151. PMC10117908 37089546

[53] D. Liu , Z. Zhang , Y. Song , et al., “Effects of Salinity on Growth, Physiology, Biochemistry and Gut Microbiota of Juvenile Grass Carp (Ctenopharyngodon idella),” Aquatic Toxicology 258, no. 1 (2023): 106482, 10.1016/j.aquatox.2023.106482. 36924593

[54] X. Li , H. Wang , A. Hisham Abdelrahman , et al., “Temperature Modulates Gut Microbiome Disruption and Resistome Enrichment in Oxytetracycline‐treated Channel Catfish (*Ictalurus punctatus*),” Microbiology Spectrum 14, no. 1 (2026): e04187–25, 10.1128/spectrum.04187-25. PMC13141892 41910137

[55] Z. B. Wu , Q. Q. Zhang , X. H. Wang , and A. H. Li , “Alterations and Resilience of Intestinal Microbiota to Increased Water Temperature Are Accompanied by the Recovery of Immune Function in Nile Tilapia,” Scientific Reports 15, no. 1 (2025): 1421, 10.1038/s41598-025-15124-w. PMC11814331 39934152

[56] C. Zhou , S. Yang , W. Ka , et al., “Association of Gut Microbiota with Metabolism in Rainbow Trout under Acute Heat Stress,” Frontiers in Microbiology 13 (2022): 846336, 10.3389/fmicb.2022.846336. PMC9007319 35432278

[57] C. Zhou and F. Ding , “Analysis of the Alterations in Symbiotic Microbiota and Their Correlation with Intestinal Metabolites in Rainbow Trout (*Oncorhynchus mykiss*) under Heat Stress Conditions,” Animals 15, no. 14 (2025): 2017, 10.3390/ani15142017. PMC12291889 40723480

[58] H. Abdallah , J. N. Idenyi , A. D. Adeyemi , et al., “Vegetable Oil Blends and Environmental Temperature Influence the Nutrient Efficiency and Gut Microbiome of Rainbow Trout: Insight on Microbial Diversity, Community Structure, and Function,” Nature Communications 15, no. 1 (2024): 2334, https://www.nature.com/articles/s41467‐024‐46372‐y.

[59] R.‐X. Li , E. Amenyogbe , Y. Lu , J.‐H. Jin , R.‐T. Xie , and J.‐S. Huang , “Effects of Low‐temperature Stress on Serum Biochemical Indicators, Intestinal Microbiome, and Transcriptome of Juvenile golden Pompano (Trachinotus ovatus),” Aquaculture International 32, no. 5 (2024): 5551–5578, 10.1007/s10499-024-01511-y.

[60] S. Ma , Y. Lv , L. Hou , et al., “Effect of Acute Temperature Stress on Energy Metabolism, Immune Performance and Gut Microbiome of Largemouth Bass (Micropterus salmoides),” Aquaculture and Fisheries 10, no. 2 (2025): 260–270, 10.1016/j.aaf.2024.01.004.

[61] S. K. Ghosh , M. K. S. Wong , S. Hyodo , S. Goto , and K. Hamasaki , “Temperature Modulation Alters the Gut and Skin Microbial Profiles of Chum Salmon (*Oncorhynchus keta*),” Frontiers in Marine Science 9, no. 1 (2022): 911124, 10.3389/fmars.2022.911124.

[62] K. Steiner , O. Laroche , S. P. Walker , and J. E. Symonds , “Effects of Water Temperature on the Gut Microbiome and Physiology of Chinook Salmon (*Oncorhynchus tshawytscha*) Reared in a Freshwater Recirculating System,” Aquaculture 560, no. 1 (2022): 738529, 10.1016/j.aquaculture.2022.738555.

[63] R. Wang , Y. Li , Z. He , et al., “Changes of Intestinal Microbiota and Metabolites in *Huso dauricus* under Heat Stress and Their Potential Relationship with Heat Stress Resistance,” Aquaculture Reports 47, no. 1 (2026): 103439, 10.1016/j.aqrep.2026.103211.

[64] S. Yang , C. Zhang , W. Xu , et al., “Heat Stress Decreases Intestinal Physiological Function and Facilitates the Proliferation of Harmful Intestinal Microbiota in Sturgeons,” Frontiers in Microbiology 13 (2022): 755369, 10.3389/fmicb.2022.755369. PMC8959899 35356512

[65] C. E. Reid , R. S. Taylor , A. Bissett , B. F. Nowak , and J. P. Bowman , “Characterization of Bacteria Colonizing the Mucosal Layer of the Gastrointestinal Tract of Atlantic Salmon Farmed in a Warm Water Region,” Frontiers in Microbiology 16, no. 1 (2025): 1421094, 10.3389/fmicb.2025.1421094. PMC12327903 40771687

[66] H. Sun , F. Chen , W. Zheng , et al., “Impact of Captivity and Natural Habitats on Gut Microbiome in *Epinephelus Akaara* across Seasons,” BMC Microbiology 24, no. 1 (2024): 114, 10.1186/s12866-024-03211-w. PMC11221007 38961321

[67] S. C. Debnath , D. L. Chaput , J. McMurtrie , et al., “Seasonal Dynamics and Factors Shaping Microbiomes in Freshwater Finfish Earthen Aquaculture Ponds in Bangladesh,” Environmental Microbiome 20, no. 1 (2025): 15, 10.1186/s40793-025-00612-x. PMC11960027 40165346

[68] M. Fang , Z. Lei , M. Ruilin , W. Jing , and D. Leqiang , “High Temperature Stress Induced Oxidative Stress, Gut Inflammation and Disordered Metabolome and Microbiome in Tsinling Lenok Trout,” Ecotoxicology and Environmental Safety 266, no. 1 (2023): 115607, 10.1016/j.ecoenv.2023.115589. 37862746

[69] Y. Sun , Y. Yan , S. Yan , et al., “Prevalence, Antibiotic Susceptibility, and Genomic Analysis of *Vibrio Alginolyticus* Isolated From Seafood and Freshwater Products in China,” Frontiers in Microbiology 15, no. 1 (2024): 1381457, 10.3389/fmicb.2024.1381457. PMC11266014 39050630

[70] L. Bai , Z. Wang , H. Wang , and B. Ma , “Comprehensive Evaluation of Environment Adaptability in Wild and Captive Lenok (*Brachymystax lenok*): From the Perspective of Antioxidant Capacity, Immune Response and Gut Microbiome,” Frontiers in Microbiology 17, no. 1 (2026): 153211, 10.3389/fmicb.2026.153211. PMC12996260 41859446

[71] X. Liangliang , H. Chen , L. Zhao , et al., “Host Habitats Influence the Gut Microbial Assembly Mechanisms of Cold‐water Fish,” Aquaculture Reports 36, no. 1 (2024): 102094, 10.1016/j.aqrep.2024.102094.

[72] C. Heys , B. Cheaib , A. Busetti , et al., “Neutral Processes Dominate Microbial Community Assembly in Atlantic Salmon, *Salmo salar* ,” Applied and Environmental Microbiology 86, no. 8 (2020), 10.1128/AEM.02283-19. PMC7117918 32033945

[73] N. K. Bereded , G. B. Abebe , S. W. Fanta , et al., “The Impact of Sampling Season and Catching Site (Wild and Aquaculture) on Gut Microbiota Composition and Diversity of Nile Tilapia (*Oreochromis niloticus*),” Biology 10, no. 3 (2021): 211, 10.3390/biology10030211. PMC8001861 33804538

[74] G. Montalvo‐Fernández , J. M. Ortiz‐Alcantara , C. Durruty‐Lagunes , et al., “Composition and Structure of Gut Microbiota of Wild and Captive *Epinephelus morio* via 16S rRNA Analysis and Functional Prediction,” Microorganisms 13, no. 8 (2025): 1654, 10.3390/microorganisms13081654. PMC12388694 40871296

[75] M. Ozen and E. C. Dinleyici , “The History of Probiotics: The Untold Story,” Beneficial Microbes 6, no. 2 (2015): 159–165, 10.3920/bm2014.0103. 25576593

[76] T. Vasiljevic and N. P. Shah , “Probiotics—From Metchnikoff to Bioactives,” International Dairy Journal 18, no. 7 (2008): 714–728, 10.1016/j.idairyj.2008.03.004.

[77] T. Al Thuwaini , M. B. S. Al‐Shuhaib , O. T. Aljumaili , et al., “Genetic Diversity and Population Structure of Iraqi Sheep Breeds Using Mitochondrial DNA Markers,” Animals 15, no. 20 (2025): 2986, 10.3390/ani15202986.

[78] A. Sachdeva , T. Tomar , T. Malik , A. Bains , and A. Karnwal , “Exploring Probiotics as a Sustainable Alternative to Antimicrobial Growth Promoters: Mechanisms and Benefits in Animal Health,” Frontiers in Sustainable Food Systems 8, no. 1 (2025): 1523678, 10.3389/fsufs.2024.1523678.

[79] R. J. de Cano and G. García Menéndez , “Why Clinical Trials of Microbiome Targeted Interventions Often Fail to Support Health Claims: a Commentary on Probiotics and Translational Design,” Microorganisms 14, no. 2 (2026): 470, 10.3390/microorganisms14020470. PMC12942760 41753756

[80] The Lancet Gastroenterology & Hepatology , “Probiotics: Elixir or Empty Promise?,” The Lancet Gastroenterology & Hepatology 4, no. 2 (2019): 81, 10.1016/S2468-1253(18)30415-1. 30647011

[81] R. García Cano and G. G. Menéndez , “Probiotics and Their Postbiotic Metabolites: Mechanisms Underlying Their Antimicrobial and Immunomodulatory Activities,” Probiotics and Antimicrobial Proteins 16, no. 3 (2024): 412–425, 10.1007/s12602-024-10290-8.

[82] M. P. Arena , G. Caggianiello , D. Fiocco , et al., “Barley Beta‐glucans Containing Food Enhances Probiotic Performances of Beneficial Bacteria,” Food Microbiology 39, no. 1 (2014): 1–6, 10.1016/j.fm.2013.03.010. PMC3958897 24562330

[83] H. Kim , J. Lee , and S. Park , “Probiotic Supplementation Has Sex‐dependent Effects on Immune Responses in Association with the Gut Microbiota in Community‐dwelling Older Adults: a Randomized, Double‐blind, Placebo‐controlled, Multicenter Trial,” Nutrition Research and Practice 17, no. 5 (2023): 883–895, 10.4162/nrp.2023.17.5.883. PMC10522805 37780220

[84] U. Wendel , “Assessing Viability and Stress Tolerance of Probiotics—A Review,” Frontiers in Microbiology 12, no. 1 (2022): 818468, 10.3389/fmicb.2021.818468. PMC8829321 35154042

[85] M. S. García , D. L. Molina , A. M. Martín Sánchez , and A. M. Cazares , “Characterization of Lactic Acid Bacteria Isolated From Fermented Foods and Evaluation of Their Probiotic Potential,” Annals of Microbiology 75, no. 1 (2025): Article 18, 10.1186/s13213-025-01825-7.

[86] P. De Vos , G. M. Garrity , and D. Jones , Bergey's Manual of Systematic Bacteriology: Volume 3: The Firmicutes, 2nd ed. (Springer, 2009), 10.1007/978-0-387-68489-5.

[87] G. Løkka , L. Austbø , K. Falk , I. Bjerkås , and E. O. Koppang , “Intestinal Morphology of the Wild Atlantic Salmon (*Salmo salar*),” Journal of Morphology 274, no. 8 (2013): 859–876, 10.1002/jmor.20142. 23520065

[88] K. M. Stehfest , C. G. Carter , J. D. McAllister , J. D. Ross , and J. M. Semmens , “Response of Atlantic Salmon *Salmo salar* to Temperature and Dissolved Oxygen Extremes Established Using Animal‐Borne Environmental Sensors,” Scientific Reports 7, no. 1 (2017): 4545, 10.1038/s41598-017-04806-2. PMC5495760 28674437

[89] N. Nimalan , S. L. Sørensen , A. Feckaninová , et al., “Supplementation of Lactic Acid Bacteria Has Positive Effects on the Mucosal Health of Atlantic Salmon (*Salmo salar*) Fed Soybean Meal,” Aquaculture Reports 28 (2023): 101461, 10.1016/j.aqrep.2022.101461.

[90] E. D. Linton , B. Jónsson , and D. L. G. Noakes , “Effects of Water Temperature on the Swimming and Climbing Behaviour of Glass Eels, Anguilla spp,” Environmental Biology of Fishes 78, no. 3 (2007): 189–192, 10.1007/s10641-005-1367-9.

[91] M. Carda‐Diéguez , R. Ghai , F. Rodriguez‐Valera , and C. Amaro , “Metagenomics of the Mucosal Microbiota of European Eels,” Genome announcements 2, no. 6 (2014): e01132–14, 10.1128/genomea.01132-14. PMC4223461 25377710

[92] P. Zhu , M. K.‐S. Wong , X. Lin , et al., “Changes of the Intestinal Microbiota along the Gut of Japanese Eel ( Anguilla japonica ),” Letters in Applied Microbiology 73, no. 4 (2021): 529–541, 10.1111/lam.13539. 34265084

[93] H. M. Abdel‐Latif , N. Abdel‐Razek , and R. H. Khalil , “Comparative Efficacy of *Bacillus* Probiotics and Formalin‐Killed Bacterin against *Vibrio Anguillarum* in European Eel Elvers,” Scientific Reports 16, no. 1 (2026): 4367, 10.1038/s41598-026-35298-8. PMC12864994 41629546

[94] S. Natale , F. Moroni , R. Domingo‐Bretón , and C. Amaro , “Dietary Lipid Utilisation under Different Thermal Conditions in Flathead Grey Mullet (*Mugil cephalus*),” Frontiers in Marine Science 13 (2026): 1847546, 10.3389/fmars.2026.1847546.

[95] E. Gisbert , S. Molas , E. Hernández , R. Carbó , and A. Ruiz , “Production of Flathead Grey Mullet (*Mugil cephalus*) and Lettuce (*Lactuca sativa*) in a Coupled Aquaponic System under Suboptimal Water Temperatures,” Fishes 9, no. 6 (2024): 189, 10.3390/fishes9060189.

[96] J. García‐Márquez , I. M. Cerezo , F. L. Figueroa , et al., “First Evaluation of Associated Gut Microbiota in Wild Thick‐Lipped Grey Mullets (*Chelon labrosus*, Risso 1827),” Fishes 7, no. 4 (2022): 209, 10.3390/fishes7040209.

[97] R. Floris , G. Sanna , C. T. Satta , et al., “Microbial Communities Associated with the Intestinal Tract of Grey Mullets from a Mediterranean Aquatic Environment,” Int J Aquac Fish Sci 10 (2024): 9–19, 10.17352/2455-8400.000091.

[98] C.‐H. Chan , L.‐H. Chen , K.‐Y. Chen , et al., “Single‐Strain Probiotics Enhance Growth, Anti‐Pathogen Immunity, and Resistance to *Nocardia Seriolae* in Grey Mullet (*Mugil cephalus*) via Gut Microbiota Modulation,” Animal Microbiome 6, no. 1 (2024): 67, 10.1186/s42523-024-00353-0. PMC11575433 39563419

[99] M. Calcagnile , S. M. Tredici , and P. Alifano , “A Comprehensive Review on Probiotics and Their Use in Aquaculture: Biological Control, Efficacy, and Safety through the Genomics and Wet Methods,” Heliyon 10, no. 24 (2024): e40892, 10.1016/j.heliyon.2024.e40892. PMC11681891 39735631

[100] Y. Zhang , H. Li , X. Wang , et al., “Effects of Temperature and Lactic Acid Bacteria Additives on the Fermentation Quality and Microbial Community of Whole‐plant Corn Silage,” BMC Plant Biology 24 (2024): Article 5501, 10.1186/s12870-024-05501-x. PMC11382506 39251915

[101] L. Zhang , Y. Li , X. Guo , et al., “The Anti‐inflammatory Effect of *Lactobacillus Plantarum* on LPS‐induced Macrophage Inflammation through the TLR4/NF‐κB Signaling Pathway,” Biomedicine & Pharmacotherapy 111 (2019): 105–114, 10.1016/j.biopha.2018.12.104.

[102] K. Thakur , B. Singh , S. Kumar , et al., “Potential of Probiotics and Postbiotics in Aquaculture: Connecting Current Research Gaps and Future Perspectives,” The Microbe 8, no. 1 (2025): 100431, 10.1016/j.microb.2025.100431.

[103] G. Vinderola , M. E. Sanders , S. Salminen , and H. Szajewska , “Postbiotics: the Concept and Their Use in Healthy Populations,” Frontiers in Nutrition 9 (2022): 1002213, 10.3389/fnut.2022.1002213. PMC9780264 36570166

[104] J. Morán and A. Kilasoniya , “Integration of Postbiotics in Food Products through Attenuated Probiotics: a Case Study with Lactic Acid Bacteria in Bread,” Foods 13 (2024): 2042, 10.3390/foods13132042. PMC11240946 38998548

[105] G. Vinderola , C. Druart , L. Gosálbez , S. Salminen , N. Vinot , and S. Lebeer , “Postbiotics in the Medical Field under the Perspective of the ISAPP Definition: Scientific, Regulatory, and Marketing Considerations,” Frontiers in Pharmacology 14 (2023): 1239745, 10.3389/fphar.2023.1239745. PMC10515206 37745060

[106] M. Moradi , R. Molaei , and J. T. Guimarães , “A Review on Preparation and Chemical Analysis of Postbiotics from Lactic Acid Bacteria,” Enzyme and Microbial Technology 143, no. 1 (2021): 109722, 10.1016/j.enzmictec.2020.109722. 33375981

[107] N. Salazar , M. Gueimonde , A. M. Hernández‐Barranco , et al., “Exopolysaccharides Produced by Intestinal *Bifidobacterium* Strains Act as Fermentable Substrates for Human Intestinal Bacteria,” Applied and Environmental Microbiology 82, no. 7 (2016): 1956–1968, 10.1128/AEM.03964-15. PMC2519331 18539803

[108] C. Sequeiros , M. E. Garcés , M. Vallejo , E. R. Marguet , and N. L. Olivera , “Potential Aquaculture Probiont *Lactococcus Lactis* TW34 Produces Nisin Z and Inhibits the Fish Pathogen *Lactococcus Garvieae* ,” Archives of Microbiology 197, no. 3 (2014): 449–458, 10.1007/s00203-014-1076-x. 25549984

[109] M. K. Yadav , J. H. Song , R. Vasquez , J. S. Lee , H. Kim , and D.‐K. Kang , “Methods for Detection, Extraction, Purification, and Characterization of Exopolysaccharides of Lactic Acid Bacteria: a Systematic Review,” Foods 13, no. 22 (2024): 3687, 10.3390/foods13223687. PMC11594216 39594102

[110] N. L. Rose , P. Sporns , and L. M. McMullen , “Detection of Bacteriocins by Matrix‐Assisted Laser Desorption/Ionization Time‐of‐Flight Mass Spectrometry,” Applied and Environmental Microbiology 65, no. 5 (1999): 2238–2242, 10.1128/AEM.65.5.2238-2242.1999. PMC91324 10224027

[111] R. Lv , B. Li , Y. Xiao , et al., “Lipopeptide‐producing *Bacillus* sp. JK08,” Journal of Applied Microbiology 134, no. 4 (2023): lxad084, 10.1093/jambio/lxad084. 37081765

[112] D. Saiyam , A. Dubey , M. A. Malla , and A. Kumar , “Lipopeptides from Bacillus: Unveiling Biotechnological Prospects—Sources, Properties, and Diverse Applications,” Brazilian Journal of Microbiology 55, no. 1 (2024): 281–295, 10.1007/s42770-023-01228-3. PMC10920585 38216798

[113] M. Geissler , C. Oellig , K. Moss , W. Schwack , M. Henkel , and R. Hausmann , “High‐performance Thin‐layer Chromatography (HPTLC) for the Simultaneous Quantification of the Cyclic Lipopeptides Surfactin, Iturin A and Fengycin in Culture Samples of *Bacillus* Species,” Journal of Chromatography B 1044‐1045, no. 1 (2017): 214–224, 10.1016/j.jchromb.2016.11.013. 28153674

[114] X. Y. Gao , Y. Liu , L. L. Miao , E.‐W. Li , T.‐T. Hou , and Z.‐P. Liu , “Mechanism of Anti‐Vibrio Activity of Marine Probiotic Strain *Bacillus Pumilus* H2, and Characterization of the Active Substance,” AMB Express 7, no. 1 (2017): 23, 10.1186/s13568-017-0323-3. PMC5241254 28097594

[115] D. Wang , J. Li , G. Zhu , et al., “Mechanism of the Potential Therapeutic Candidate *Bacillus Subtilis* BSXE‐1601 against Shrimp Pathogenic Vibrios and Multifunctional Metabolites Biosynthetic Capability of the Strain as Predicted by Genome Analysis,” Frontiers in Microbiology 11, no. 1 (2020): 581802, 10.3389/fmicb.2020.581802. PMC7649127 33193216

[116] Y.‐A. Chen , W.‐C. Chiu , T.‐Y. Wang , H.‐C. Wong , and C.‐T. Tang , “Isolation and Characterization of an Antimicrobial *Bacillus Subtilis* Strain O‐741 against *Vibrio Parahaemolyticus* ,” PLoS ONE 19, no. 4 (2024): 0299015, 10.1371/journal.pone.0299015. PMC10994408 38573920

[117] C. M. Calvanese , F. Villani , D. Ercolini , and F. De Filippis , “Postbiotics versus Probiotics: Possible New Allies for Human Health,” Food Research International 217, no. 1 (2025): 116869, 10.1016/j.foodres.2025.116869. 40597563

[118] H. S. Kusuma , T. Widyastuti , N. Rahmawati , and D. K. Sari , “Analysis of Energy‐peaks Characteristic in NaI(Tl) Spectrometer for Radionuclide Identification in Environmental Radiation Monitoring Device,” Journal of Physics: Conference Series 1528, no. 1 (2020): 012015, 10.1088/1755-1315/462/1/012015.

[119] Y. Hirose , S. Murosaki , T. Fujiki , Y. Yamamoto , Y. Yoshikai , and M. Yamashita , “Lipoteichoic Acids on *Lactobacillus Plantarum* Cell Surfaces Correlate with Induction of Interleukin‐12p40 Production,” Microbiology and Immunology 54, no. 3 (2010): 143–151, 10.1111/j.1348-0421.2009.00189.x. 20236424

[120] L. Alvarez , S. B. Hernandez , M. A. de Pedro , and F. Cava , “Ultra‐Sensitive, High‐Resolution Liquid Chromatography Methods for the High‐Throughput Quantitative Analysis of Bacterial Cell Wall Chemistry and Structure,” Bacterial Cell Wall Homeostasis: Methods in Molecular Biology 1440, no. 1 (2016): 11–25, 10.1007/978-1-4939-3676-2_2. 27311661

[121] E. M. Anderson , N. A. Greenwood , D. Brewer , and C. M. Khursigara 1 , “Semi‐Quantitative Analysis of Peptidoglycan by Liquid Chromatography Mass Spectrometry and Bioinformatics,” Journal of Visualized Experiments 164, no. 1 (2020): 61799, 10.3791/61799. 33135689

[122] Y. Zhong , Z. Wang , H. Xie , et al., “The Immunostimulatory Effects of CpG Oligodeoxynucleotides in Fish: a Review,” Journal of Fish Diseases 35, no. 11 (2012): 812–825, 10.1111/j.1365-2761.2012.01423.x.

[123] D.‐W. Yeh , Y.‐L. Liu , Y.‐C. Lo , et al., “Toll‐Like Receptor 9 and 21 Have Different Ligand Recognition Profiles and Cooperatively Mediate Activity of CpG‐oligodeoxynucleotides in Zebrafish,” Proceedings of the National Academy of Sciences 110, no. 51 (2013): 20711–20716, 10.1073/pnas.1305273110. PMC3870712 24282308

[124] C.‐Y. Lai , G.‐Y. Yu , Y. Luo , R. Xiang , and T.‐H. Chuang , “Immunostimulatory Activities of CpG‐Oligodeoxynucleotides in Teleosts: Toll‐Like Receptors 9 and 21,” Frontiers in Immunology 10, no. 1 (2019): 179, 10.3389/fimmu.2019.00179. PMC6375897 30800129

[125] M. Xie , Y. Li , R. E. Olsen , et al., “Dietary Supplementation of Exopolysaccharides From *Lactobacillus Rhamnosus* GCC‐3 Improved the Resistance of Zebrafish against Spring Viremia of Carp Virus Infection,” Frontiers in Immunology 13, no. 1 (2022): 968348, 10.3389/fimmu.2022.968348. PMC9389081 35990638

[126] M. S. R. Rajoka , Y. Wu , H. M. Mehwish , M. Bansal , and L. Zhao , “Lactobacillus Exopolysaccharides: New Perspectives on Engineering Strategies, Physiochemical Functions, and Immunomodulatory Effects on Host Health,” Trends in Food Science & Technology 103, no. 1 (2020): 36–48, 10.1016/j.tifs.2020.06.003.

[127] K. Pandey , G. Prakash TV , H. Gosai , D. Bhatia , and R. Patel , “Microbial Exopolysaccharides as Postbiotics: Structure, Function, and Translational Potential,” International Journal of Biological Macromolecules 330, no. Pt 1 (2025): 147956, 10.1016/j.ijbiomac.2025.147956. 41027519

[128] Y. Cao , M. Xu , Q. Chen , D. Wu , J. Lu , and G. Cai , “Potential Nutritional and Functional Matters in Yeast Culture Prepared by Soybean Meal Fermentation,” Journal of the Science of Food and Agriculture 104, no. 14 (2024): 8869–8878, 10.1002/jsfa.13713. 38963133

[129] G. Paglia and G. Astarita , “A High‐Throughput HILIC‐MS‐Based Metabolomic Assay for the Analysis of Polar Metabolites,” Methods in Molecular Biology 2396, no. 1 (2022): 185–198, 10.1007/978-1-0716-1822-6_11. 34786681

[130] K. Mohammad , H. Jiang , and V. I. Titorenko , “Quantitative Metabolomics of *Saccharomyces Cerevisiae* Using Liquid Chromatography Coupled with Tandem Mass Spectrometry,” Journal of Visualized Experiments no. 167 (2021): 62061, 10.3791/62061. 33491678

[131] L. Spaggiari , G. Tedeschi , G. Benatti , et al., “Untargeted Metabolomic Analysis of Cell‐Free Supernatants (CFSs) from Different Clinical Isolates of *Saccharomyces Cerevisiae* and Their Effects on *Candida albicans* Virulence,” Journal of Fungi 12, no. 2 (2026): 81, 10.3390/jof12020081. PMC12941470 41745224

[132] H.‐S. Son , G.‐S. Hwang , K. M. Kim , et al., “1H NMR‐Based Metabolomic Approach for Understanding the Fermentation Behaviors of Wine Yeast Strains,” Analytical Chemistry 81, no. 3 (2009): 1137–1145, 10.1021/ac802305c. 19115855

[133] S. Guglielmetti , M. Boyte , and C. Smith , “Commercial and Regulatory Frameworks for Postbiotics: an Industry‐Oriented Scientific Perspective for Non‐Viable Microbial Ingredients Conferring Beneficial Physiological Effects,” Trends in Food Science & Technology 163, no. 1 (2025), Article 105130, 10.1016/j.tifs.2025.105130.

[134] D. Merenstein , B. Pot , G. Layer , et al., “Emerging Issues in Probiotic Safety: 2023 Perspectives,” Gut Microbes 15, no. 1 (2023): 2185034, 10.1080/19490976.2023.2185034. PMC10026873 36919522

[135] P. Liu , Y. Liu , J. Cheng , Y. Xia , and Y. Yang , “Copper Exposure Causes Alteration in the Intestinal Microbiota and Metabolites in *Takifugu Rubripes* ,” Ecotoxicology and Environmental Safety 272, no. 1 (2024): 116064, 10.1016/j.ecoenv.2024.116064. 38340599

[136] M. Vass , A. Abramova , and J. Bengtsson‐Palme , “Antimicrobial Resistance Dissemination via Horizontal Gene Transfer Is Constrained in Stratified Waters,” Communications Biology 9, no. 1 (2026): 435, 10.1038/s42003-026-09857-8. PMC13022341 41813906

[137] K. Abe , N. Nomura , and S. Suzuki , “Biofilms: Hot Spots of Horizontal Gene Transfer (HGT) in Aquatic Environments, with a Focus on a New HGT Mechanism,” FEMS Microbiology Ecology 96, no. 5 (2020): fiaa031, 10.1093/femsec/fiaa031. PMC7189800 32109282

[138] E. Wangkahart , P. T. Lee , and C. M. Chong , “Antimicrobial Resistance (AMR) in Farmed Aquatic Organisms,” in Antimicrobial Resistance in Aquaculture and Aquatic Environments, ed. P. Elumalai and S. Lakshmi (Springer, 2025), 65–88, 10.1007/978-981-97-7320-6_4.

[139] S. H. Hoseinifar , Y.‐Z. Sun , and C. M. A. Caipang , “Short Chain Fatty Acids as Feed Supplements for Sustainable Aquaculture: an Updated Review,” Aquaculture Reports 21, no. 1 (2021): 100784, 10.1016/j.aqrep.2021.100784.

[140] M. A. Anokyewaa , Z. Wang , E. Amenyogbe , Y. Lu , and G. Zhen , “Impacts of Probiotics on Microbial Populations in Aquaculture Systems,” Microbial Ecology 89, no. 1 (2026): 94, 10.1007/s00248-026-02710-9. PMC13056774 41882188

[141] X. Shen , H. Jin , F. Zhao , L.‐Y. Kwok , Z. Zhao , and Z. Sun , “Short‐term Probiotic Supplementation Affects the Diversity, Genetics, Growth, and Interactions of the Native Gut Microbiome,” iMeta 3, no. 6 (2024): 253, 10.1002/imt2.253. PMC11683461 39742297

[142] C. Giraud , N. Wabete , C. Lemeu , et al., “Environmental Factors and Potential Probiotic Lineages Shape the Active Prokaryotic Communities Associated with Healthy *Penaeus Stylirostris* Larvae and Their Rearing Water,” FEMS Microbiology Ecology 100, no. 12 (2024): fiae156, 10.1093/femsec/fiae156. PMC11636268 39562288

[143] EFSA Panel on Additives and Products or Substances used in Animal Feed (FEEDAP) , “Safety and Efficacy of *Lactobacillus Plantarum* DSM 21762 as a Feed Additive for all Animal Species,” EFSA Journal 18, no. 3 (2020): 6174, 10.2903/j.efsa.2020.6174.

[144] J. G. Wang , M. T. Lei , Y. Liu , Z. Wang , J. Fan , and Y. Wang , “Insights From Antibiotic‐induced Gut Microbiota Dysbiosis and Metabolic Changes in the Gut and Hypothalamus into the Microbiota‐gut‐brain Axis in Large Yellow Croaker (*Larimichthys crocea*),” Aquaculture 599, no. 1 (2025): 741812, 10.1016/j.aquaculture.2024.741812.

[145] Z. Zhang , J. Lv , L. Pan , et al., “Roles and Mechanisms of Probiotics in Regulating Host Health,” Probiotics and Antimicrobial Proteins 12 (2020): 1024–1040, 10.1007/s12602-020-09633-y.

[146] J. Suez , T. Korem , D. Zeevi , et al., “Artificial Sweeteners Induce Glucose Intolerance by Altering the Gut Microbiota,” Proceedings of the Royal Society B: Biological Sciences 283, no. 1821 (2016): 20153106, 10.1098/rspb.2015.3069.

[147] Z. Zhang , D. Li , M. Rehman , et al., “Characterization of Lactic Acid Bacteria Isolated From Fermented Vegetables and Their Potential Probiotic Properties,” Scientific Reports no. 6 (2016): 23214, 10.1038/srep23214.

[148] Y. Chen , X. Li , L. Wang , et al., “Effects of Probiotics on Growth, Immunity, and Gut Microbiota in Children: a Systematic Review and Meta Analysis,” Children 8 (2021): 924, 10.3390/children8100924.

[149] J. Suez and E. Elinav , “The Role of the Microbiome in Health and Disease: an Overview,” Progress in Molecular Biology and Translational Science 190 (2022): 1–31, 10.1016/bs.pmbts.2022.07.003.

[150] R. C. Robertson , A. R. Manges , B. B. Finlay , and A. J. Prendergast , “The Human Microbiome and Child Growth—First 1000 Days and Beyond,” Trends in Microbiology 27, no. 2 (2019): 131–147, 10.1016/j.tim.2018.09.008. 30529020

[151] G. F. R. Mogoș , A. Chiracu , M. Mitran , et al., “Intestinal Microbiota in Early Life: Latest Findings Regarding the Role of Probiotics as a Treatment Approach for Dysbiosis,” Nutrients 17, no. 13 (2025): 2071, 10.3390/nu17132071. PMC12250897 40647176

[152] J. Kim , H. Lee , and S. Park , “Probiotic Modulation of Host Immunity: Molecular Mechanisms and Therapeutic Potential,” BMB Reports 57, no. 2 (2024): 123–134, 10.5483/BMBRep.2023-0114.

[153] G. Vinderola , M. E. Sanders , M. Cunningham , and C. Hill , “Frequently Asked Questions about the ISAPP Postbiotic Definition,” Frontiers in Microbiology 14, no. 1 (2024): 1324565, 10.3389/fmicb.2023.1324565. PMC10807003 38268705

[154] J. E. Aguilar‐Toalá , R. Garcia‐Varela , H. S. Garcia , et al., “Postbiotics: an Evolving Term within the Functional Foods Field,” Trends in Food Science & Technology 75, no. 1 (2018): 105–114, 10.1016/j.tifs.2018.03.009.

[155] J. Żółkiewicz , A. Marzec , M. Ruszczyński , and W. Feleszko , “Postbiotics—A Step beyond Prebiotics and Probiotics,” Nutrients 12, no. 8 (2020): 2189, 10.3390/nu12082189. PMC7468815 32717965

[156] P. F. Cuevas‐González , A. M. Liceaga , and J. E. Aguilar‐Toalá , “Postbiotics and Paraprobiotics: From Concepts to Applications,” Food Research International 136, no. 1 (2020): 109502, 10.1016/j.foodres.2020.109502. 32846581

[157] V. Taverniti and S. Guglielmetti , “The Immunomodulatory Properties of Probiotic Microorganisms beyond Their Viability (ghost probiotics: Proposal of paraprobiotic concept),” Genes & Nutrition 6, no. 3 (2011): 261–274, 10.1007/s12263-011-0218-x. PMC3145061 21499799

[158] C. N. de Almada , R. C. R. Martinez , and A. S. Sant'Ana , “Paraprobiotics: Evidence on Biological Responses and Applications in Foods,” Trends in Food Science & Technology 58, no. 1 (2016): 96–114, 10.1016/j.tifs.2016.09.011.

[159] J. Xie , B. Li , Y. Xiao , et al., “Isolation and Characterization of a Lipopeptide‐producing *Bacillus* sp. Strain JK08 with Antimicrobial Field Properties,” Journal of Chemical Technology & Biotechnology 98, no. 11 (2023): 2514–2523, 10.1002/jctb.7431.

[160] T. Wu , X. Hu , W. Xu , Y. Du , and J. Chen , “Effect of Dietary Supplement of Inactivated Lactobacillus Plantarum Ep‐M17 on Growth Performance, Immune Response, Disease Resistance, and Intestinal Microbiota in Penaeus vannamei,” Journal of Oceanology and Limnology 42, no. 2 (2024): 676–694, 10.1007/s00343-023-2399-8.

[161] G. Vinderola , A. Benkowski , M. Bernardeau , et al., “Postbiotics: a Perspective on Their Quantification,” Frontiers in Nutrition 12, no. 1 (2025): 1582733, 10.3389/fnut.2025.1582733. PMC12175706 40535053

[162] S. D. C. Rocha , P. Lei , B. Morales‐Lange , L. T. Mydland , and M. Øverland , “From a Cell Model to a Fish Trial: Immunomodulatory Effects of Heat‐Killed *Lactiplantibacillus Plantarum* as a Functional Ingredient in Aquafeeds for Salmonids,” Frontiers in Immunology 14, no. 1 (2023): 1125702, 10.3389/fimmu.2023.1125702. PMC10040762 36993984

[163] A. Gonçalves , A. Amiot , and P. Langella , “Bacterial Extracellular Vesicles as Emerging Postbiotics,” Trends in Food Science & Technology 110, no. 1 (2021): 308–316, 10.1016/j.tifs.2021.02.001.

[164] J. R. Tagg , A. S. Dajani , and L. W. Wannamaker , “Bacteriocins of Gram‐positive Bacteria,” Bacteriological Reviews 40, no. 3 (1976): 722–756, 10.1128/br.40.3.722-756.1976. PMC413978 791239

[165] M. A. Riley and J. E. Wertz , “Bacteriocins: Evolution, Ecology, and Application,” Annual Review of Microbiology 56, no. 1 (2002): 117–137, 10.1146/annurev.micro.56.012302.161042. 12142491

[166] P. D. Cotter , C. Hill , and R. P. Ross , “Bacteriocins: Developing Innate Immunity for Food,” Nature Reviews Microbiology 3, no. 10 (2005): 777–788, 10.1038/nrmicro1273. 16205711

[167] P. A. Wescombe , N. C. Heng , J. P. Burton , C. N. Chilcott , and J. R. Tagg , “Streptococcal Bacteriocins and the Case for Streptococcus Salivarius as Model Oral *Probiotics* ,” Future Microbiology 4, no. 7 (2009): 819–835, 10.2217/fmb.09.61. 19722837

[168] V. Pybus , M. W. Loutit , I. L. Lamont , and J. R. Tagg , “Growth Inhibition of the Salmon Pathogen *Vibrio Ordalii* by a Siderophore Produced by *Vibrio Anguillarum* Strain VL4355,” Journal of Fish Diseases 17, no. 4 (1994): 311–324, 10.1111/j.1365-2761.1994.tb00227.x.

[169] B. Austin , L. F. Stuckey , P. A. W. Robertson , I. Effendi , and D. R. W. Griffith , “A Probiotic Strain of *Vibrio Alginolyticus* Effective in Reducing Diseases Caused by *Aeromonas Salmonicida*, *Vibrio Anguillarum* and *Vibrio Ordalii* ,” Journal of Fish Diseases 18, no. 1 (1995): 93–96, 10.1111/j.1365-2761.1994.tb00227.x.

[170] F. H. Yildiz and K. L. Visick , “ *Vibrio* Biofilms: So Much the Same yet so Different,” Trends in Microbiology 17, no. 3 (2009): 109–118, 10.1016/j.tim.2008.12.004. PMC2729562 19231189

[171] W. Chu , S. Zhou , W. Zhu , and X. Zhuang , “Quorum Quenching Bacteria Bacillus sp. QSI‐1 Protect Zebrafish (Danio rerio) From Aeromonas Hydrophila Infection,” Scientific Reports 4, no. 1 (2014): 5446, 10.1038/srep05446. PMC4069686 24962441

[172] L. Monzón‐Atienza , J. Bravo , S. Torrecillas , et al., “An in‐Depth Study on the Inhibition of Quorum Sensing by Bacillus Velezensis D‐18: Its Significant Impact on Vibrio Biofilm Formation in Aquaculture,” Microorganisms 12, no. 5 (2024): 890, 10.3390/microorganisms12050890. PMC11123725 38792721

[173] N. T. Tran , W. Yang , X. T. Nguyen , et al., “Application of Heat‐killed Probiotics in Aquaculture,” Aquaculture 548, no. 1 (2022): 737700, 10.1016/j.aquaculture.2021.737700.

[174] L.‐T. Tao , H. Lu , J. Xiong , L. Zhang , W.‐W. Sun , and X.‐F. Shan , “The Application and Potential of Postbiotics as Sustainable Feed Additives in Aquaculture,” Aquaculture 592, no. 1 (2024): 741237, 10.1016/j.aquaculture.2024.741237.

[175] E. Torres‐Maravilla , M. Parra , K. Maisey , et al., “Importance of Probiotics in Fish Aquaculture: towards the Identification and Design of Novel Probiotics,” Microorganisms 12, no. 3 (2024): 626, 10.3390/microorganisms12030626. PMC10975645 38543677

[176] B. Vallejo‐Cordoba , C. Castro‐López , H. S. García , et al., “Postbiotics and Paraprobiotics: a Review,” Advances in Food and Nutrition Research 94, no. 1 (2020): 1–34, 10.1016/bs.afnr.2020.06.001. 32892831

[177] S. R. Elsegeny , F. S. Radwan , Y. M. Elshamy , et al., “A Comprehensive Overview of Probiotics in Aquaculture: From Efficacy Evaluation to Diverse Applications,” Annals of Microbiology 75, no. 1 (2025): 35, 10.1186/s13213-025-01825-7.

[178] G. Vinderola , M. E. Sanders , and S. Salminen , “The Concept of Postbiotics,” Foods 11, no. 8 (2022): 1077, 10.3390/foods11081077. PMC9027423 35454664

[179] Z. Cheng , Q. Wang , K. Lei , et al., “Positive Effects of Dietary Postbiotics on Growth, Intestinal Health, Immunity, and Ammonia Nitrogen Tolerance in Hybrid Grouper (*Epinephelus fuscoguttatus* ♀ × *Epinephelus lanceolatus* ♂),” Comparative Immunology Reports 10, no. 1 (2025): 200264, 10.1016/j.cirep.2025.200264.

[180] K. R. Leistikow , E. B. Rachelle , and K. R. Hristova , “Probiotics beyond the Farm: Benefits, Costs, and Considerations of Using Antibiotic Alternatives in Livestock,” Frontiers in Antibiotics 1 (2022): 1003912, 10.3389/frabi.2022.1003912. PMC11732145 39816405

[181] M. Domínguez‐Maqueda , J. García‐Márquez , S. T. Tapia‐Paniagua , et al., “Evaluation of the Differential Postbiotic Potential of *Shewanella Putrefaciens* Pdp11 Cultured in Several Growing Conditions,” Marine Biotechnology 26, no. 1 (2024): 1–18, 10.1007/s10126-023-10271-y. PMC10869407 38153608

[182] H. Liu , H. Zhang , Q. Yu , et al., “Lead Induced Structural and Functional Damage and Microbiota Dysbiosis in the Intestine of Crucian Carp (*Carassius auratus*),” Frontiers in Microbiology 14, no. 1 (2023): 118421, 10.3389/fmicb.2023.118421. PMC10507410 37731918

[183] M. Domínguez‐Maqueda , J. García‐Márquez , S. T. Tapia‐Paniagua , et al., “Evaluation of the Differential Postbiotic Potential of *Shewanella Putrefaciens* Pdp11 Cultured in Several Growing Conditions,” Marine Biotechnology 26, no. 1 (2024): 1–18, 10.1007/s10126-023-10271-y. PMC10869407 38153608

[184] M. Khakpour , M. Mohsenzadeh , and A. Salari , “Feasibility of *Lactiplantibacillus Plantarum* Postbiotics Production in Challenging Media by Different Techniques,” AMB Express 14, no. 1 (2024): 47, 10.1186/s13568-024-01704-5. PMC11052967 38668839

[185] L. Brown , J. M. Wolf , R. Prados‐Rosales , and A. Casadevall , “Through the Wall: Extracellular Vesicles in Gram‐Positive Bacteria, Mycobacteria and Fungi,” Nature Reviews Microbiology 13, no. 10 (2015): 620–630, 10.1038/nrmicro3480. PMC4860279 26324094

[186] M. Kumar , W. M. Devi , T. G. Choudhury , et al., “Unraveling the Bioactivities and Immunomodulatory Potential of Postbiotics Derived From *Bacillus Subtilis* and *B. amyloliquefaciens* for Aquaculture,” Probiotics and Antimicrobial Proteins 18, no. 2 (2026): 338–352, 10.1007/s12602-025-10528-z. 40186049

[187] Z.‐Y. Liu , H.‐L. Yang , S. Li , et al., “Paraprobiotic and Postbiotic Forms of *Bacillus Siamensis* Improved Growth, Immunity, Liver and Intestinal Health in *Lateolabrax Maculatus* Fed Soybean Meal Diet,” Fish & Shellfish Immunology 145, no. 1 (2024): 109370, 10.1016/j.fsi.2024.109370. 38216004

[188] F. Jafarzadeh , L. Roomiani , M. C. Dezfoulnejad , M. J. Baboli , and A. A. Sary , “Harnessing Paraprobiotics and Postbiotics for Enhanced Immune Function in Asian Seabass (*Lates calcarifer*): Insights into Pattern Recognition Receptor Signaling,” Fish & Shellfish Immunology 151, no. 1 (2024): 109725, 10.1016/j.fsi.2024.109725. 38925448

[189] B. Liang and D. Xing , “The Current and Future Perspectives of Postbiotics,” Probiotics and Antimicrobial Proteins 15, no. 6 (2023): 1626–1643, 10.1007/s12602-023-10045-x. PMC9913028 36763279

[190] C. Yao Ang , M. Sano , S. Dan , M. Leelakriangsak , and T. M. Lal , “Postbiotics Applications as Infectious Disease Control Agents in Aquaculture,” Biocontrol Science 25, no. 1 (2020): 1–7, 10.4265/bio.25.1. 32173662

[191] M. L. Chikindas , D. E. Roopchand , S. K. Tiwari , et al., “An Integrated Engineering Approach to Creating Health‐Modulating Postbiotics,” Molecular Nutrition & Food Research 70, no. 1 (2026): 70326, 10.1002/mnfr.70326. PMC12728392 41251175

[192] J. R. Bradley , “TNF‐Mediated Inflammatory Disease,” Cytokine & Growth Factor Reviews 19, no. 3–4 (2008): 187–191, 10.1016/j.cyto.2007.11.006.

[193] N. R. Sproston and J. J. Ashworth , “Role of C‐reactive Protein,” International Journal of Molecular Sciences 19, no. 6 (2018): 1714, 10.3390/ijms19061814.

[194] C. A. Hunter and S. A. Jones , “IL‐6 as a Keystone Cytokine in Health and Disease,” Nature Reviews Immunology 15, no. 8 (2015): 523–534, 10.1038/nri3717. 25898198

[195] N. Valdés , A. Romero , and L. Mercado , “Environmental Factors Affecting Immune Responses in Aquaculture,” Aquaculture 490, no. 1 (2018): 1–12, 10.1016/j.aquaculture.2018.02.037.

[196] S. H. Hoseinifar , E. Ringø , H. Van Doan , et al., “Potential of Probiotics and Postbiotics in Aquaculture: a Comprehensive Review,” Regional Studies in Marine Science 75, no. 1 (2025): 1001992, 10.1016/j.rsma.2025.1001992.

[197] M. Quintanilla‐Pineda , C. G. Achou , J. Díaz , et al., “In Vitro Evaluation of Postbiotics Produced From Bacterial Isolates Obtained From Rainbow Trout and Nile Tilapia against the Pathogens *Yersinia Ruckeri* and *Aeromonas Salmonicida* subsp. *Salmonicida* ,” Foods 12, no. 4 (2023): 861, 10.3390/foods12040861. PMC9957526 36832935

[198] H. M. R. Abdel‐Latif , A. A. Soliman , H. Sewilam , et al., “The Influence of Dietary Postbiotics on the Growth Performance, Protozoan Counts, and Antioxidant Status of Nile Tilapia (*Oreochromis niloticus*),” Aquaculture Reports 18, no. 1 (2020): 100551, 10.1016/j.aqrep.2020.100551.

[199] M. C. Guimarães , I. M. Cerezo , M. F. Fernandez‐Alarcon , et al., “Oral Administration of Probiotics (*Bacillus subtilis* and *Lactobacillus plantarum*) in Nile Tilapia (*Oreochromis niloticus*) Vaccinated and Challenged with *Streptococcus agalactiae* ,” Fishes 7, no. 4 (2022): 211, 10.3390/fishes7040211.

[200] S. Agriopoulou , E. Stamatelopoulou , and T. Varzakas , “Microbiome Engineering to Enhance Disease Resistance in Aquaculture: Integration of Probiotics and Postbiotics,” Frontiers in Microbiology 16, no. 1 (2025): 1625265, 10.3389/fmicb.2025.1625265. PMC12479497 41035886

[201] M. A. O. Dawood , S. Koshio , M. El‐Sabagh , et al., “A Novel Postbiotic Product Based on *Weissella cibaria* for Rainbow Trout (*Oncorhynchus mykiss*): Effects on Growth, Immunity, and Disease Resistance,” Animals 14, no. 5 (2024): 744, 10.3390/ani14050744. PMC10930836 38473129

[202] R. Ballantyne , J.‐W. Lee , S.‐T. Wang , et al., “Dietary Administration of a Postbiotic, Heat‐killed Pediococcus Pentosaceus PP4012 Enhances Growth Performance, Immune Response and Modulates Intestinal Microbiota of White Shrimp, Penaeus vannamei, Heat‐Killed *Pediococcus Pentosaceus* PP4012 Enhances Growth Performance, Immune Response and Modulates Intestinal Microbiota of White Shrimp (*Penaeus vannamei*),” Fish & Shellfish Immunology 139, no. 1 (2023): 108882, 10.1016/j.fsi.2023.108882. 37279829

[203] J. Xiong , K. Wang , J. Wu , et al., “Changes in Intestinal Bacterial Communities Are Closely Associated with Shrimp Disease Severity,” Applied Microbiology and Biotechnology 99, no. 16 (2015): 6911–6919, 10.1007/s00253-015-6632-z. 25947250

[204] M. Muneeb , A. S. Alawam , S. Batool , et al., “Nutritional and Functional Feed Additives in Fishes: a Comprehensive Review,” Aquaculture Reports 48, no. 1 (2026): 103615, 10.1016/j.aqrep.2026.103615, Article.

[205] I. Marmelo , M. Dias , A. Grade , et al., “Immunomodulatory and Antioxidant Effects of Functional Aquafeeds Biofortified with Whole *Laminaria Digitata* in Juvenile Gilthead Seabream (*Sparus aurata*),” Frontiers in Marine Science 11, no. 1 (2024): 1325244, 10.3389/fmars.2024.1325244, Article.

[206] Z. Abdul Kari , “Nutritional Immunomodulation in Aquaculture: Functional Nutrients, Stress Resilience, and Sustainable Health Strategies,” Aquaculture International 33, no. 6 (2025): 441, 10.1007/s10499-025-02122-5.

[207] T. Pérez , J. L. Balcázar , I. Ruiz‐Zarzuela , et al., “Host–Microbiota Interactions within the Fish Intestinal Ecosystem,” Mucosal Immunology 3, no. 4 (2010): 355–360, 10.1038/mi.2010.12. 20237466

[208] C. C. Lazado and C. M. A. Caipang , “Mucosal Immunity and Probiotics in Fish,” Fish & Shellfish Immunology 39, no. 1 (2014): 78–89, 10.1016/j.fsi.2014.04.015. 24795079

[209] S. Hatano , Y. Hirose , Y. Yamamoto , S. Murosaki , and Y. Yoshikai , “Scavenger Receptor for Lipoteichoic Acid Is Involved in the Potent Ability of *Lactobacillus Plantarum* Strain L‐137 to Stimulate Production of Interleukin‐12p40,” International Immunopharmacology 25, no. 2 (2015): 321–331, 10.1016/j.intimp.2015.02.011. 25698554

[210] Y. Hirose , S. Murosaki , Y. Yamamoto , Y. Yoshikai , and T. Tsuru , “Daily Intake of Heat‐Killed *Lactobacillus Plantarum* L‐137 Augments Acquired Immunity in Healthy Adults,” The Journal of Nutrition 136, no. 12 (2006): 3069–3073, 10.1093/jn/136.12.3069. 17116721

[211] S. Murosaki , Y. Yamamoto , K. Ito , et al., “Heat‐killed Lactobacillus Plantarum L‐137 Suppresses Naturally Fed Antigen–specific IgE Production by Stimulation of IL‐12 Production in Mice,” Journal of Allergy and Clinical Immunology 102, no. 1 (1998): 57–64, 10.1016/s0091-6749(98)70055-7. 9679848

[212] X. Li , T. Han , S. Zheng , and G. Wu , “Hepatic Glucose Metabolism and Its Disorders in Fish,” Advances in Experimental Medicine and Biology 1354, no. 1 (2022): 207–236, 10.1007/978-3-030-85686-1_11. 34807444

[213] P. Enes , S. Panserat , S. Kaushik , and A. Oliva‐Teles , “Growth Performance and Metabolic Utilization of Diets with Increasing Levels of Carbohydrates in Fish,” Comparative Biochemistry and Physiology Part A: Molecular & Integrative Physiology 158, no. 3 (2011): 247–251, 10.1016/j.cbpa.2011.01.001.

[214] H. S. Yang , “Lipid Biomarkers and Cardiometabolic Diseases: Critical Knowledge Gaps and Future Research Directions,” Metabolites 15, no. 2 (2025): 108, 10.3390/metabo15020108. PMC11857555 39997733

[215] L. Tóthová , O. Nagy , and H. Seidel , “Blood Biochemical Markers in Fish: a Review,” Veterinarni Medicina 61, no. 10 (2016): 537–552, 10.17221/46/2016-VETMED.

[216] J. Hu , J. Zuo , J. Li , et al., “Effects of Secondary Polyethylene Microplastic Exposure on Crucian (*Carassius carassius*) Growth, Liver Damage, and Gut Microbiome Composition,” Science of the Total Environment 802, no. 1 (2022): 149736, 10.1016/j.scitotenv.2021.149736. 34464809

[217] M. J. García Mansilla , A. Vallejo‐García , M. Sánchez , et al., “Microbial Derived Antioxidants in Intestinal Inflammation: a Systematic Review of Their Therapeutic Potential,” Antioxidants 14, no. 3 (2025): 321, 10.3390/antiox14030321. PMC11939483 40227262

[218] S. Agriopoulou , E. Stamatelopoulou , and T. Varzakas , “Postbiotics: the New Frontier in Food and Health,” Foods 9, no. 9 (2020): 1184, 10.3390/foods9091184.

[219] Y. Zhu , H. Yang , X. Liu , et al., “Effects of Postbiotics on Intestinal Health,” Journal of Animal Science and Biotechnology 13, no. 1 (2022): 14, 10.1186/s40104-021-00662-w.

[220] D. Pismennyi , W. D. Jamieson , N. Tufenkji , et al., “Measurement of Peptidoglycan Fragments,” Microorganisms 11 (2023): 2134, 10.3390/microorganisms11092134.

[221] I. M. Cerezo , O. Pérez‐Gómez , S. Rohra‐Benítez , M. Domínguez‐Maqueda , J. García‐Márquez , and S. Arijo , “Postbiotics of Marine Origin and Their Therapeutic Application,” Marine Drugs 23, no. 9 (2025): 335, 10.3390/md23090499. PMC12471580 41003304

[222] M. Quintanilla‐Pineda , F. C. Ibañez , C. C. Garrote‐Achou , and F. Marzo , “Profiling a New Postbiotic Product,” Fishes 8, no. 6 (2023): 304, 10.3390/fishes8060304.

[223] K. Y. Ko , S. R. Park , and M. Kim , “Analysis Method for Determination of Nisin A and Nisin Z in Cow Milk by Using Liquid Chromatography‐tandem Mass Spectrometry,” Journal of Dairy Science 98, no. 3 (2015): 1435–1442, 10.3168/jds.2014-8452. 25529415

[224] X. Wu , T. Teame , Q. Hao , et al., “Use of a Paraprobiotic and Postbiotic Feed Supplement (HWF™) Improves the Growth Performance, Composition and Function of Gut Microbiota in Hybrid Sturgeon (Acipenser baerii x Acipenser schrenckii),” Fish & Shellfish Immunology 104, no. 1 (2020): 36–45, 10.1016/j.fsi.2020.05.054. 32473360

[225] S. Fanning , L. J. Hall , M. Cronin , et al., “Bifidobacterial Surface‐exopolysaccharide Facilitates Commensal‐host Interaction through Immune Modulation and Pathogen Protection,” Proceedings of the National Academy of Sciences 109, no. 6 (2012): 2108–2113, 10.1073/pnas.1115621109. PMC3277520 22308390

[226] L. W. Sumner , A. Amberg , D. Barrett , et al., “Proposed Minimum Reporting Standards for Chemical Analysis,” Metabolomics 3, no. 3 (2007): 211–221, 10.1007/s11306-007-0082-2. PMC3772505 24039616

[227] M. Quintanilla‐Pineda , F. C. Ibañez , C. C. Garrote‐Achou , and F. Marzo , “Postbiotic Based on *Weissella cibaria* ,” Animals 14, no. 5 (2024): 744, 10.3390/ani14050744. PMC10930836 38473129

[228] A. K. Warda , P. H. de Almeida Bettio , C. M. Hueston , G. Di Benedetto , A. G. Clooney , and C. Hill , “Oral Administration of Heat‐Treated Lactobacilli Modifies the Murine Microbiome and Reduces Citrobacter Induced Colitis,” Frontiers in Microbiology 11, no. 1 (2020): 69, 10.3389/fmicb.2020.00069. PMC7003559 32082288

[229] L. Guo , X. Ze , H. Feng , et al., “Identification and Quantification of Viable *Lacticaseibacillus Rhamnosus* in Probiotics Using Validated PMA‐qPCR Method,” Frontiers in Microbiology 15, no. 1 (2024): 1341884, 10.3389/fmicb.2024.1341884. PMC10828034 38298895

[230] X. Peng , A. Ed‐Dra , and M. Yue , “Whole Genome Sequencing for the Risk Assessment of Probiotic Lactic Acid Bacteria,” Critical Reviews in Food Science and Nutrition 63, no. 32 (2023): 11244–11262, 10.1080/10408398.2022.2087174. 35694810

[231] C. Araújo , E. Muñoz‐Atienza , T. Pérez‐Sánchez , et al., “Nisin Z Production by *Lactococcus Lactis* subsp. *Cremoris* WA2‐67 of Aquatic Origin as a Defense Mechanism to Protect Rainbow Trout (*Oncorhynchus mykiss*, Walbaum) against *Lactococcus Garvieae* ,” Marine Biotechnology 17, no. 6 (2015): 820–830, 10.1007/s10126-015-9660-x. 26307018

[232] D. Pismennõi , A. Kattel , I. Belouah , R. Nahku , R. Vilu , and E.‐G. Kobrin , “The Quantitative Measurement of Peptidoglycan Components Obtained from Acidic Hydrolysis in Gram‐Positive and Gram‐Negative Bacteria via Hydrophilic Interaction Liquid Chromatography Coupled with Mass Spectrometry,” Microorganisms 11, no. 9 (2023): 2134, 10.3390/microorganisms11092134. PMC10534856 37763978

[233] S. Wuertz , A. Schroeder , and K. M. Wanka , “Probiotics in Fish Nutrition—Long‐Standing Household Remedy or Native Nutraceuticals?,” Water 13, no. 10 (2021): 1348, 10.3390/w13101348.

[234] Y. Gao , L. Qiang , N. Wu , et al., “Study on the Potential Probiotics Isolated from Marine Aquaculture System and Evaluation for Aquaculture Application,” Aquaculture Research 2024, no. 1 (2024): 9555271, 10.1155/2024/9555271.

[235] S. Katariya , K. Khapandi , A. Gadhiya , and D. Chhatrodiya , “Marine‐Derived Probiotic Microorganisms: a Review of Their Therapeutic Applications in Human Health,” The Microbe 10 (2026): 100680, 10.1016/j.microb.2026.100680.

[236] S. E. Gilliland and D. Walker , “Factors to Consider When Selecting a Culture of *Lactobacillus Acidophilus* as a Dietary Adjunct to Produce a Hypocholesterolemic Effect in Humans,” Journal of Dairy Science 81, no. 12 (1998): 2591–2599, 10.3168/jds.S0022-0302(98)75549-3. 2111831

[237] M. A. A. Sumon , S. J. Khan , M. J. Islam , et al., “Epigenetics and Postbiotics in Aquaculture: Unlocking Sustainable Solutions through Innovation,” Fish & Shellfish Immunology 166, no. 1 (2025): 110598, 10.1016/j.fsi.2025.110598. 40721123

[238] M. S. Hossain , M. M. Rahman , M. A. Islam , et al., “Probiotics and Their Role in Modulating Gut Microbiota: Implications for Health and Disease,” Microorganisms 14, no. 6 (2024): 1230, 10.3390/microorganisms14061230.

[239] M. S. Hossain , M. M. Rahman , M. A. K. Parvez , et al., “Probiotics and Their Role in Modulating Gut Microbiota: Implications for Health and Disease,” Microbial Pathogenesis 195, no. 1 (2025): 100431, 10.1016/j.micpath.2025.100431.

[240] Z. A. Nomi , “Postbiotics from Agro‐Industrial Waste: Sustainable Bioactive Production for Food and Health Application,” in Animal Health and Disease Management (Pioneer Page Publishers, 2024), 165–172, 10.5281/zenodo.17171852.

[241] V. Paralika and P. Makridis , “Microbial Interactions in Rearing Systems for Marine Fish Larvae,” Microorganisms 13, no. 3 (2025): 539, 10.3390/microorganisms13030539. PMC11945982 40142430

[242] G. Radhamanalan , “Postbiotics and Phage Synergy in Precision Oral Microbiome Engineering: Systems Biology Strategies Targeting *Streptococcus Mutans* in Dental Caries,” Frontiers in Molecular Biosciences 13, no. 1 (2026): 1766853, 10.3389/fmolb.2026.1766853. PMC13099311 42028131

[243] D. Han , Y. Zhang , W. Liu , et al., “Novel Antimicrobial Strategy: Native Postbiotics Synergize with Antibiotics to Combat Klebsiella pneumoniae,” BMC Microbiology 25 (2025): 4747, 10.1186/s12866-025-04474-7. PMC12604375 41214548

[244] R. L. Helm , B. L. Jutras , T. G. DeHart , S. Hildreth , and W. K. Ray , “Muropeptide LC‐MS Analysis and Data Processing Protocols for Core Facilities,” Journal of Biomolecular Techniques Suppl (2020): S17, 10.7171/jbt.20-3104-003.

